# Macrophage Derived Galectin‐3 Promotes Renal Fibrosis and Diabetic Kidney Disease by Enhancing TGFβ1 Signaling

**DOI:** 10.1002/advs.202504032

**Published:** 2025-08-13

**Authors:** Yibing Chen, Qian Jiang, Xiaowei Xing, Liu Xu, Qijin Zhao, Qidong Zhang, Jingwen Chen, Chunxiao Ma, Xingfeng Liu, Yinfei Lu, Yanjun Wan, Lijuan Kong, Shaocong Hou, Qian Lu, Bing Cui, Jiankan Guo, Hongdong Huang, Pingping Li

**Affiliations:** ^1^ State Key Laboratory of Bioactive Substance and Function of Natural Medicines, Institute of Materia Medica Chinese Academy of Medical Sciences and Peking Union Medical College Beijing 100050 China; ^2^ Jiangsu Key Laboratory of New Drug Research and Clinical Pharmacy Xuzhou Medical University Xuzhou 221004 China; ^3^ Department of Nephrology, Beijing Friendship Hospital Capital Medical University Beijing 100034 China; ^4^ Internal Medicine/Section of Nephrology Yale University New Haven CT 06520 US

**Keywords:** DKD, galectin‐3, protein degradation, renal fibrosis, TGFβ1 signaling

## Abstract

Over 30% of patients with type 2 diabetes develop diabetic kidney disease (DKD), which has emerged as a major contributor to end stage renal disease. Renal fibrosis represents the final pathological outcome of most chronic kidney disease, particularly DKD. This study demonstrates elevated levels of Galectin‐3 (Gal3), a lectin associated with inflammatory and fibrotic conditions, in the plasma and kidneys of DKD mice. Positive correlations between Gal3 expression and renal fibrosis are observed in both DKD patients and mice. Macrophage‐derived Gal3 is found to promote Transforming growth factor beta 1 (TGFβ1) signaling activation and renal fibrogenesis. Genetic ablation of Gal3 globally or specifically in macrophages, as well as pharmacological inhibition of Gal3, significantly attenuated kidney fibrosis in diabetic mice. Mechanistically, macrophage‐derived Gal3 interacted with TGFβ receptor2 (TGFBR2) and Pro‐TGFβ1, preventing TGFBR2 proteasomal degradation in fibroblasts and increasing TGFβ1 levels in the diabetic kidney. These events enhances TGFβ1 signaling activation and ultimately facilitated kidney fibrosis. The findings of this study suggest Gal3 as a potential therapeutic target for renal fibrosis and DKD.

## Graphical abstract

Macrophage‐derived Galectin‐3 amplifies TGFβ1 signaling to drive diabetic kidney fibrosis: a novel therapeutic target.

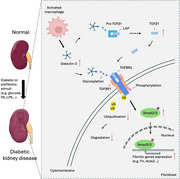

## 1 Introduction

Diabetic kidney disease (DKD), a prevalent form of chronic kidney disease (CKD), is a major contributor to end‐stage renal disease.^[^ ^1^ ^]^ It affects around 30% of type 1 and 40% of type 2 diabetes patients.^[^ ^2^, ^3^ ^]^ With the increasing incidence of diabetes, the incidence of DKD is also on the rise.^[^ ^4^ ^]^ The pathogenesis of DKD remains complex and incompletely understood. It is thought that during the progression of diabetes, hyperglycemia and dyslipidemia induce metabolic disturbances in renal cells and alter plasma hemodynamics in renal micro‐vessels. These changes initiate inflammatory and fibrotic responses, leading to renal damage.^[^ ^5^, ^6^ ^]^ If uncontrolled, continuous activation of inflammatory and fibrotic pathways exacerbates kidney injury, promotes extracellular matrix accumulation, and ultimately results in renal fibrosis,^[^ ^5^ ^]^ a common outcome of DKD.^[^ ^7^ ^]^ Currently, effective treatments for renal fibrosis and DKD are limited.

Galectin‐3 (Gal3) is a β‐galactoside‐binding lectin with widespread expression, involved in regulating diverse cellular processes, such as cell growth, proliferation, differentiation, inflammation, and fibrosis.^[^ ^8^, ^9^ ^]^ Elevated plasma and urine Gal3 levels have been reported in patients with DKD, and renal biopsies suggest that infiltration of Gal3‐positive cell is associated with a poor prognosis in DKD patients.^[^ ^10^, ^11^, ^12^, ^13^, ^14^, ^15^ ^]^ Pharmacological inhibition of Gal3 has been shown to mitigate the progression of hypertensive kidney disease in rats.^[^ ^16^ ^]^ Gal3 is also closely linked to fibrotic diseases. Inhibition of Gal3 has demonstrated beneficial effects in myocardial fibrosis, idiopathic pulmonary fibrosis, non‐alcoholic steatohepatitis and CKD.^[^ ^17^ ^]^ Genetic ablation of Gal3 significantly improves renal fibrosis in unilateral ureteral obstruction (UUO) mice, with macrophage‐derived Gal3 playing a pivotal role.^[^ ^18^ ^]^ Additionally, kidney transplantation‐induced tubular atrophy and interstitial fibrosis are markedly alleviated in Gal3‐deficient mice.^[^ ^19^ ^]^ Despite evidence highlighting the importance of Gal3 in tissue fibrosis, the specific molecular mechanisms remain incompletely understood. The potential of Gal3 as a therapeutic target for kidney fibrosis and DKD also requires further investigation.

Transforming growth factor beta 1 (TGFβ1) is a key cytokine involved in the regulation of cellular and tissue fibrosis.^[^ ^20^ ^]^ In fibroblasts, TGFβ1 binds to TGFβ receptors (TGFBR2 and TGFBR1), activating the Small mothers against decapentaplegic (Smad2/3) signaling pathway., The activation promotes the expression of fibrosis‐related genes (e.g., *Acta2*, *Fn1*, *Col1a1*.) and the transformation of fibroblasts into myofibroblasts.^[^ ^21^, ^22^ ^]^ During the progression of DKD, pro‐fibrotic factors can enhance TGFβ1 expression and activation directly or indirectly, leading to excessive fibroblast activation, a critical step in renal fibrosis development.^[^ ^7^ ^]^ Although targeting TGFβ1 shows potential for treating renal fibrosis in DKD mice, the development of ideal strategies to interfere with TGFβ1 signaling is challenging due to its multiple and essential roles in maintaining cellular pathophysiological functions.^[^ ^23^, ^24^ ^]^ Therefore, elucidating the regulatory mechanisms of TGFβ1 signaling and developing novel therapeutic approaches for renal fibrosis and DKD are of significant importance.

This study investigated the role of Gal3 in renal fibrosis during DKD and explored the underlying mechanisms. It revealed a novel pattern by which Gal3 regulates TGFβ1 signaling in renal fibrosis, providing experimental insights for the development of therapeutic strategies for renal fibrosis and DKD.

## 2 Results

### 2.1 Increased Gal3 Levels in DKD Patients and Mice

**Figure 1:**
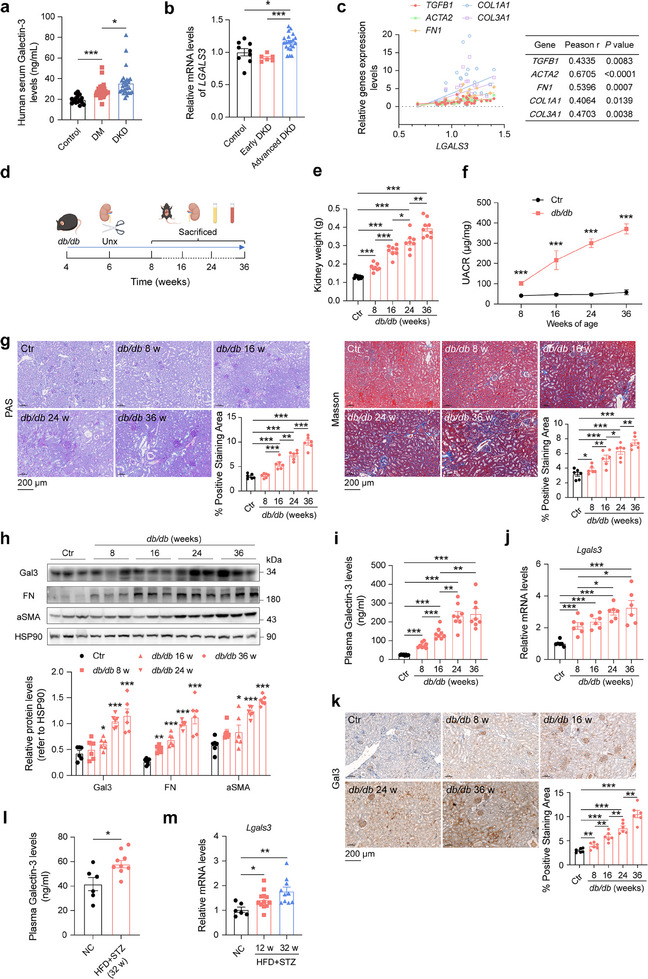
Increased serum and kidney Gal3 levels in patients and mice with DKD. a) Serum Gal3 levels in control, diabetic (DM) and DKD patients (n = 15 individuals for control, n = 29 patients for DM, n = 28 patients for DKD). b, c) Human kidney *LGALS3* mRNA levels in control, early DKD and advanced DKD patients (b) and the expression correlations of fibrotic genes (*TGFB1, ACTA2, FN1, COL1A1, COL3A1*) and *LGALS3* (c) (from GSE142025 dataset,^[^ ^26^ ^]^ n = 9 patients for control, n = 6 patients for early DKD, n = 21 patients for advanced DKD). d) Schematic diagram of experiment. e, f) Kidney weight e) and urine albumin creatinine ratio (UACR) f) of *db/db* mice at different weeks of age (n = 7–9 mice for each group). g) PAS and Masson staining of *db/db* mice kidney (n = 6 mice for each group, scale bar 200 µm). h) Protein expression levels of Gal3, FN and aSMA in the kidney of *db/db* mice (n = 6 mice for each group). i, j) Plasma (i) and kidney (j) Gal3 levels in *db/db* mice (n = 7–9 mice for each group). k) Immunohistochemistry (IHC) staining of Gal3 in the kidney of *db/db* mice. n = 6 mice for each group, scale bar 200 µm. l) Plasma Gal3 levels in normal chow (NC) and HFD+STZ mice (n = 6 mice for NC, n = 9 mice for HFD+STZ). m) Kidney *Lgals3* mRNA levels in NC and HFD+STZ mice (n = 6 mice for NC, n = 12 mice for HFD+STZ (12 w), n = 10 mice for HFD+STZ (32 w)). Data were analyzed by two‐tailed Student's t test and presented as the mean ± SEM. Pearson correlation coefficient was used to analyze gene expression correlations. * *P* < 0.05; ** *P* < 0.01; *** *P* < 0.001; compared with control mice or indicated groups.

To confirm the correlation between Gal3 and DKD, serum Gal3 levels were measured in diabetic patients with and without DKD. Compared to healthy controls, serum Gal3 levels were significantly elevated in diabetic patients without DKD, and were further increased in those with DKD (**Figure** **1**a). Correlation analysis revealed that higher serum Gal3 levels in DKD patients were associated with decreased estimated glomerular filtration rate (eGFR) and elevated serum creatinine levels (Figure S1a and b, Supporting Information). Reanalysis of a publicly available clinical kidney RNA‐seq dataset (GSE142025)^[^ ^25^ ^]^ revealed that kidney mRNA levels of *LGALS3*, encoding Gal3, were dramatically upregulated in patients with advanced DKD compared to controls or those with early‐stage DKD (Figure 1b). Moreover, the expressions of fibrosis‐associated genes, including *TGFB1*, *ACTA2*, *FN1*, *COL1A1*, and *COL3A1*, showed positive correlations with *LGALS3* expression (Figure 1c). These findings indicate a positive correlation between Gal3 and DKD progression. We then investigated Gal3 levels in diabetic *db/db* mice, a DKD model.^[^ ^26^, ^27^ ^]^ At 6 weeks of age, right nephrectomy was performed on *db/db* mice to accelerate kidney injury (Figure 1d). Compared to control mice, *db/db* mice exhibited higher body weight, elevated fasting blood glucose (FBG), and larger kidneys (Figure 1e; Figure S1c–e, Supporting Information). The urea albumin creatinine ratio (UACR), a key marker of kidney injury, increased steadily in *db/db* mice from 8 weeks of age (Figure 1f). Periodic acid‐Schiff (PAS) and Masson staining revealed glycogen accumulation and fibrosis in the kidneys of *db/db* mice compared to controls (Figure 1g). Additionally, the protein levels of Gal3, along with fibrosis markers such as fibronectin (FN) and α‐smooth muscle actin (aSMA), were significantly increased in *db/db* mice kidneys, accompanied by macrophage infiltration (Figure 1h,k; Figure S1f, Supporting Information). Similar to the findings in DKD patients, plasma and kidney Gal3 levels were dramatically elevated in *db/db* mice after right nephrectomy, concurrent with kidney injury and fibrosis (Figure 1i,j).

We also utilized another DKD mice model induced by high‐fat‐diet (HFD) combined with streptozocin (STZ) administration (Figure S1g, Supporting Information). In this model, mouse body weight increased after HFD feeding and decreased after STZ injection. However, FBG, kidney index (left kidney weight/body weight), and UACR levels were significantly higher in DKD mice than in normal mice (Figure S1h–k, Supporting Information). PAS and Masson staining showed increased renal fibrosis in DKD mice, which was associated with upregulated expression of renal inflammation‐ and fibrosis‐related genes (Figure S1l–n, Supporting Information). Consistent with findings in *db/db* mice and DKD patients, blood and kidney Gal3 levels were significantly increased in this DKD mouse model compared to normal mice (Figure 1l,m). These results suggest that Gal3 levels are increased in both DKD animal models and human patients.

### 2.2 Gal3 Knockout Ameliorates Kidney Fibrosis in DKD and CKD Mice

**Figure 2:**
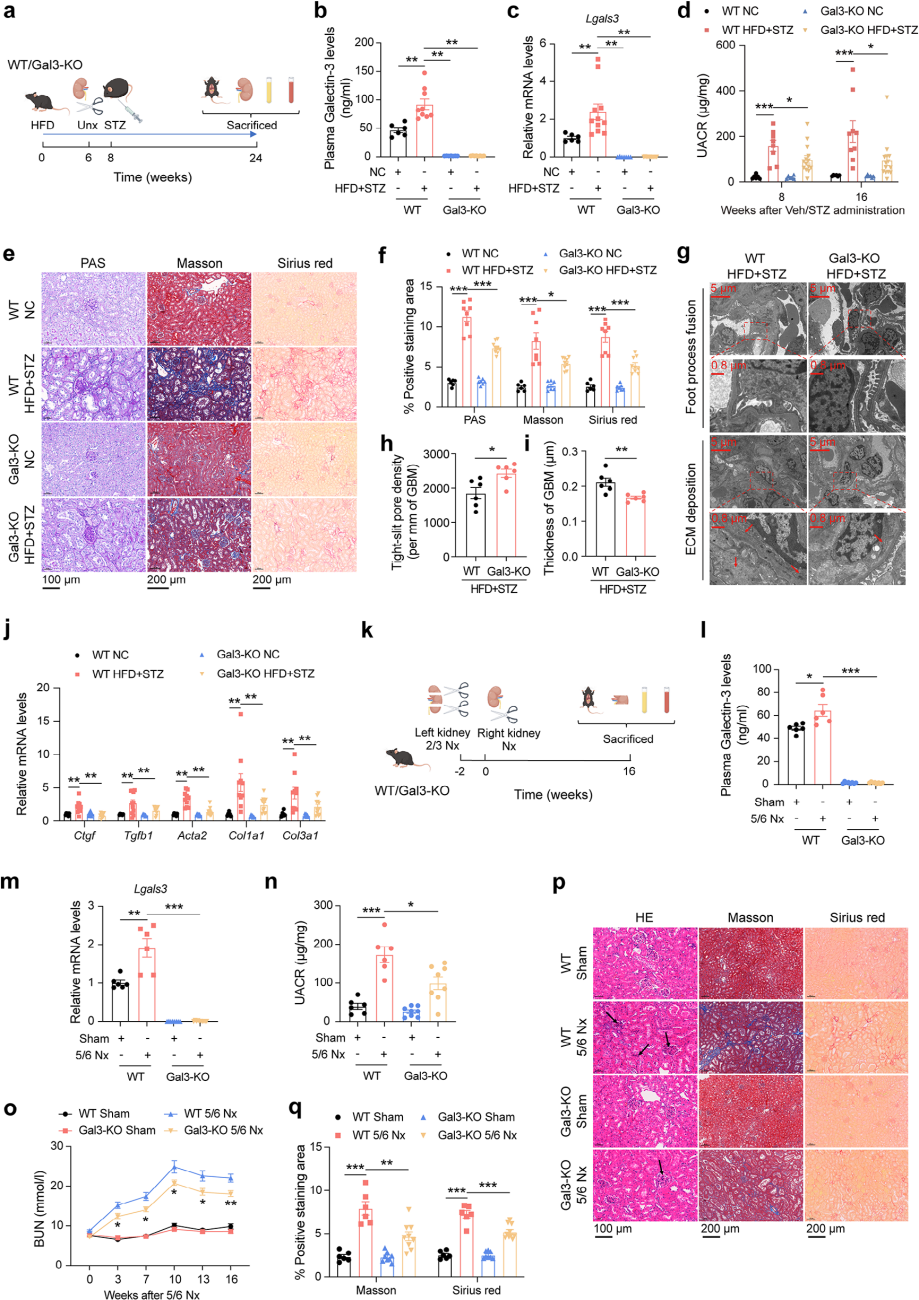
Gal3 knockout ameliorates the kidney fibrosis of DKD and CKD mice. a) Schematic diagram of experiment. b,c) Plasma (b) and kidney (c) Gal3 levels in WT and Gal3‐KO mice with or without DKD (n = 6–11 mice per group). d) UACR in WT and Gal3‐KO mice with or without DKD (n = 8–13 mice per group). e, f) PAS, Masson and Sirius red staining of kidney in WT and Gal3‐KO mice with or without DKD, representative images (e) and statistical analysis (f) (n = 6–9 mice per group, scale bar 100 µm for PAS and 200 µm for Masson and Sirius red). g–i) TEM images (g) and statistical analysis of tight‐slit pore density (h) and thickness of GBM (i) in WT and Gal3‐KO mice kidney with DKD (n = 6 mice per group, scale bar 5 µm or 0.8 µm as indicated, red arrow, ECM deposition). j) Kidney mRNA expression levels of fibrosis related genes (n = 6–11 mice per group). k) Schematic diagram of experiment. l, m) Plasma (l) and kidney (m) Gal3 levels in WT and Gal3‐KO mice with or without CKD (n = 6–10 mice per group). n‐o, UACR n) and BUN o) of WT and Gal3‐KO mice with or without CKD (n = 6–10 mice per group). p, q) HE, Masson and Sirius red staining of kidney in WT and Gal3‐KO mice with or without CKD, representative images (p) and statistical analysis (q) (n = 6–9 mice per group, scale bar 100 µm for HE and 200 µm for Masson and Sirius red, black arrow, inflammatory cell infiltration). Data were analyzed by two‐tailed Student's t test and presented as the mean ± SEM. *p < 0.05; **p < 0.01; ***p < 0.001; compared with WT CKD mice (o) or indicated groups.

To investigate the role of Gal3 in DKD development, Gal3 whole‐body knockout (Gal3‐KO) mice^[^ ^28^ ^]^ were generated. An optimized HFD combined with STZ administration protocol was used to induce DKD, aiming to exacerbate kidney injury and fibrosis (**Figure** **2**a). In DKD mice, both plasma and kidney Gal3 levels were significantly increased; these levels were undetectable following Gal3 ablation (Figure 2b,c). During DKD progression, no significant differences were observed in body weight, FBG, or kidney index between wild type (WT) and Gal3‐KO mice (Figure S2a–c, Supporting Information). However, the UACR was significantly lower in Gal3‐KO DKD mice compared with WT DKD mice (Figure 2d). PAS, Masson and Sirius red staining demonstrated that Gal3 deletion alleviated renal glycogen accumulation and fibrosis in DKD mice (Figure 2e,f). Transmission electron microscopy (TEM) was employed to assess renal microstructural damage. The results showed that podocyte foot process fusion and renal extracellular matrix (ECM) deposition were improved in Gal3‐KO DKD mice (Figure 2g–i). Moreover, the expression levels of fibrosis‐ and inflammation‐ related genes were decreased in Gal3‐KO DKD mice compared to WT DKD mice (Figure 2j; Figure S2d, Supporting Information).

Given that DKD is a subtype of CKD, we hypothesized that Gal3 may have conserved functions, such as promoting renal inflammation and fibrosis, beyond DKD. To test this, we explored the role of Gal3 in CKD pathogenesis. CKD was induced in WT and Gal3‐KO mice via 5/6 nephrectomy (Figure 2k). Similar to DKD models, both plasma and renal Gal3 levels were increased in CKD mice (Figure 2l,m). Compared to the controls, Gal3 deletion significantly ameliorated kidney function in CKD mice, as evidenced by reduced UACR and blood urea nitrogen (BUN) levels, without affecting body weight (Figure 2n,o; Figure S2e, Supporting Information). Hematoxylin and eosin (HE), Masson and Sirius red staining, along with gene expression analysis, revealed that renal inflammation and fibrosis were attenuated in Gal3‐KO CKD mice compared with WT CKD mice (Figure 2p,q; Figure S2f and g, Supporting Information).

These results suggested an important role of Gal3 in renal fibrosis. Gal3 ablation improved renal function and reduced fibrosis in both DKD and CKD mouse models, suggesting that Gal3 may be integral to common profibrotic signaling pathways.

### 2.3 Macrophage‐Derived Gal3 Induces Kidney Fibrosis and DKD Progression

**Figure 3:**
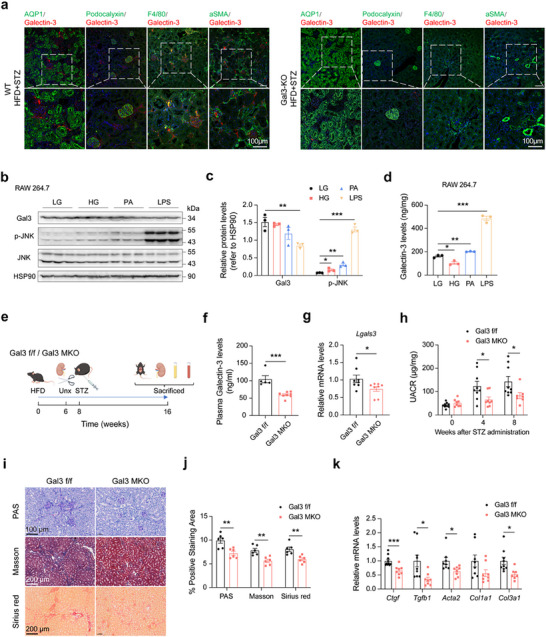
Macrophage derived Gal3 induces kidney fibrosis in DKD mice. a) Kidney immunofluorescence co‐staining of Gal3 (red) and other marker proteins (AQP1, Podocalyxin, F4/80, aSMA) (green) in WT and Gal3‐KO mice with DKD, scale bar 100 µm. b–d) Gal3 and JNK signaling pathway protein levels in RAW 264.7 macropahges (b) with statistical analysis (c) and Gal3 levels in cell medium supernatant (d) after LG, HG, PA and LPS treatments. LG, 5.56 mM glucose, HG, 30mM glucose, PA, 200 µM palmitic acid, LPS, 20 ng/mL lipopolysaccharide. e) Schematic diagram of experiment. f, g) Plasma (f) and kidney (g) Gal3 levels in Gal3 f/f and Gal3 MKO mice with DKD (n = 7–8 mice per group). h) UACR in Gal3 f/f and Gal3 MKO mice with DKD (n = 7–10 mice per group). i, j) PAS, Masson and Sirius red staining of kidney in Gal3 f/f and Gal3 MKO mice with DKD, representative images (i) and statistical analysis (j) (n = 6 mice per group, scale bar 100 µm for PAS and 200 µm for Masson and Sirius red). k) Kidney mRNA expression levels of fibrosis related genes in Gal3 f/f and Gal3 MKO mice with DKD (n = 7–10 mice per group). Data were analyzed by two‐tailed Student's t test and presented as the mean ± SEM. *p < 0.05; **p < 0.01; ***p < 0.001; compared with indicated groups.

The origin of the abnormally increased Gal3 in DKD remains unclear. To address this, we performed co‐staining of Gal3 with marker proteins of different types of kidney cells. These markers included aquaporin 1 (AQP1) for proximal renal tubular epithelial cells, Podocalyxin for glomerular podocytes, F4/80 for macrophages, and aSMA for activated fibroblasts and vascular smooth muscle cells. We found Gal3 was mainly co‐localized with F4/80 in the kidney of DKD mice (**Figure** **3**a; Figure S3a, Supporting Information).

Given that Gal3 is a secreted protein, we subsequently investigated the expression and secretion levels of Gal3 in various kidney cell types following common diabetic stimuli. This was done to identify the source of Gal3 during the development of DKD. When compared with fibroblasts (NIH/3T3), glomerular podocytes (MPC‐5), renal collecting duct cells (M‐1) and renal proximal tubular epithelial cells (HK‐2), macrophages (RAW 264.7) secreted significantly higher levels of Gal3 after exposure to diabetic stimuli, approximately 10 times more than other cell types (Figure 3b–d; Figure S3c–k, Supporting Information). Interestingly, treatment with palmitic acid (PA) and lipopolysaccharide (LPS) significantly enhanced Gal3 secretion in macrophages, while simultaneously reducing the intracellular levels of Gal3.

Furthermore, we generated macrophage Gal3‐specific knockout mice (Gal3 MKO) using the cre‐loxp system, as previously described.^[^ ^29^ ^]^ After establishing the DKD model (Figure 3e), Gal3 MKO mice exhibited significantly lower plasma and kidney Gal3 levels compared to control mice (Figure 3f,g). The body weight and FBG levels were comparable between Gal3 MKO and control (Gal3 f/f) mice, while the kidney index was lower in Gal3 MKO mice (Figure S4a–c, Supporting Information). Additionally, kidney function, fibrosis, and inflammation were notably improved in Gal3 MKO DKD mice when compared to Gal3 f/f DKD mice (Figure 3h–k; Figure S4d, Supporting Information).

Collectively, these results suggest that the ectopically expressed Gal3 is predominantly secreted by macrophages, and macrophage‐derived Gal3 plays a crucial role in inducing kidney fibrosis in DKD mice.

### 2.4 Gal3 Binds to TGFBR2 and Enhances the Activation Effect of TGFβ1 on Renal Fibroblasts

TGFβ1 signaling is pivotal in tissue fibrosis.^[^ ^20^ ^]^ Notably, kidney *Tgfb1* levels decreased in both DKD and CKD mice following Gal3 ablation (Figure 2j; Figure S2f, Supporting Information). Based on these observations, we hypothesized that Gal3 promotes renal fibrosis through regulating the TGFβ1 signaling pathway.

**Figure 4:**
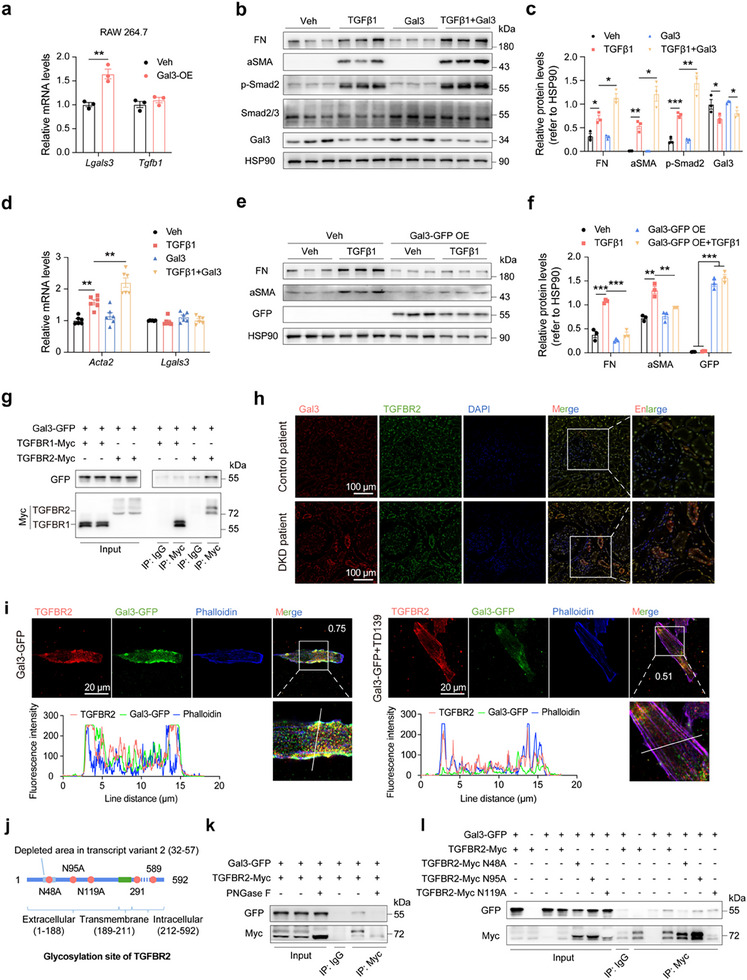
Gal3 binds to TGFBR2 and enhances the activation effect of TGFβ1 on renal fibroblast. a) *Lgals3* and *Tgfb1* mRNA levels after Gal3 overexpression in RAW 264.7 macrophages. b) Protein levels of FN, aSMA, p‐Smad2, Smad2/3 and Gal3 in NRK‐49F fibroblasts after TGFβ1 and Gal3 treatments (n = 3 independent cell samples per group). c) Statistical analysis of (b). d) mRNA levels of *Acta2* and *Lgals3* in NRK‐49F fibroblasts after TGFβ1 and Gal3 treatments (n = 6 independent cell samples per group). e) Protein levels of FN, aSMA and GFP in NRK‐49F kidney fibroblasts after Gal3‐GFP overexpression and TGFβ1 treatment (n = 3 independent cell samples per group). f) Statistical analysis of (e). g) Co‐immunoprecipitation of Gal3‐GFP and TGFBR1‐Myc and TGFBR2‐Myc in 293T cells. h) Immunofluorescence co‐staining of Gal3 (red) and TGFBR2 (green) in DKD human kidney, scale bar 100 µm. i) Immunofluorescence co‐staining of TGFBR2 (reed) and Gal3‐GFP (green) together with phalloidin in NRK‐49F fibroblast after Gal3‐GFP treatment, Pearson's r value for the colocalization of Gal3‐GFP with TGFBR2, 0.75. The co‐staining was interrupted by Gal3 inhibitor TD139 (1 µM), Pearson's r value for the colocalization of Gal3‐GFP with TGFBR2, 0.51. Scale bar 20 µm. j) Structure diagram of TGFBR2 (transcript variant 1 and variant 2), orange circle, glycosylation site. k) Co‐immunoprecipitation of Gal3‐GFP and TGFBR2‐Myc in 293T cells after PNGase F treatment. l) Co‐immunoprecipitation of Gal3‐GFP and TGFBR2‐Myc with glycosylation site mutation in 293T cells. Data were analyzed by two‐tailed Student's t test and presented as the mean ± SEM. * *p* < 0.05; ** *p* < 0.01; *** *p* < 0.001; compared with indicated groups.

Firstly, we overexpressed Gal3 in macrophages, which are the main source of *Tgfb1*. However, no significant change in *Tgfb1* expression after Gal3 overexpression (**Figure** **4**a). Similarly, overexpressing Gal3 in fibroblasts, podocytes, renal collecting duct cells, and tubular epithelial cells also did not promote *Tgfb1* expression (Figure S5a–d, Supporting Information). This suggested that the downregulated *Tgfb1* expression in the kidneys of Gal3‐knockout DKD and CKD mice might be a secondary effect. Interestingly, we found that Gal3 promoted TGFβ1‐induced renal fibroblast activation in a dose‐dependent manner (Figure S5e–g, Supporting Information). Given that Gal3 concentrations typically range from 30 to 200 ng mL^−1^ under physiological or DKD conditions, we used 200 ng mL^−1^ of Gal3 for subsequent studies. We confirmed that Gal3 enhanced the profibrotic effect of TGFβ1 in renal fibroblast, accompanied by the activation of TGFβ1 signaling (Figure 4b–d). Additionally, extracellular Gal3 had no effect on *Tgfb1* expression (Figure S5h, Supporting Information). In contrast, increasing intracellular Gal3 levels did not promote fibroblast activation; instead, it inhibited activation to some extent (Figure 4e,f). These results suggested that increased extracellular, rather than intracellular, Gal3 levels potentiated the profibrotic effect of TGFβ1 in renal fibroblasts.

It has been reported that Gal3 interacts with TGFBR2 on the cell surface of tumor cells.^[^ ^30^ ^]^ In fibroblast, TGFBR1 and TGFBR2 are the two main receptors of TGFβ1. We confirmed, through co‐immunoprecipitation, that Gal3 binds to TGFBR2 but not to TGFBR1 (Figure 4g). Human kidney biopsy results showed that, compared with the control group, there was increased co‐staining of Gal3 and TGFBR2 in patients with DKD (Figure 4h). Immunofluorescence staining indicated that Gal3 binds to TGFBR2 on the cell surface of renal fibroblast, and the Gal3 inhibitor TD139 significantly reduces this binding (Figure 4i; Figure S5i, Supporting Information).

Our previous study suggested that Gal3 can interact with glycoprotein at glycosylation site.^[^ ^29^ ^]^ TGFBR2 has three extracellular glycosylation sites (N48, N95 and N119) (Figure 4j). Further co‐immunoprecipitation experiments revealed that deglycosylation of TGFBR2 by PNGase F diminished its binding to Gal3 (Figure 4k). Point‐mutation of the extracellular glycosylation sites of TGFBR2 also reduces their interaction, with N48 and N95 being the major glycosylation sites for Gal3 binding (Figure 4l).

Additionally, we generated truncated Gal3 variants. One variant contains only the carbohydrate recognition domain (CRD) (Gal3‐CRD), and the other lacks the CRD (Gal3‐NSR, composed of the N‐terminal short end and the repeat motif region). The results demonstrated that Gal3 primarily interacts with TGFBR2 through its CRD (Figure S6d, Supporting Information). Furthermore, Gal3‐CRD, but not Gal3‐NSR, enhances the effect of TGFβ1 on FN, phosphorylated Smad2 (p‐Smad2), and aSMA, indicating that the binding of Gal3 and TGFBR2 promotes TGFβ1 signaling and fibrosis (Figure S6f and g, Supporting Information).

Taken together, we found that extracellular Gal3 promotes the activation effect of TGFβ1 on renal fibroblasts and binds to TGFBR2. These findings suggest that Gal3 may contribute to renal fibrosis through regulating TGFBR2 expression.

### 2.5 Gal3 Inhibits the Ubiquitin‐Proteasome Degradation Pathway of TGFBR2

**Figure 5:**
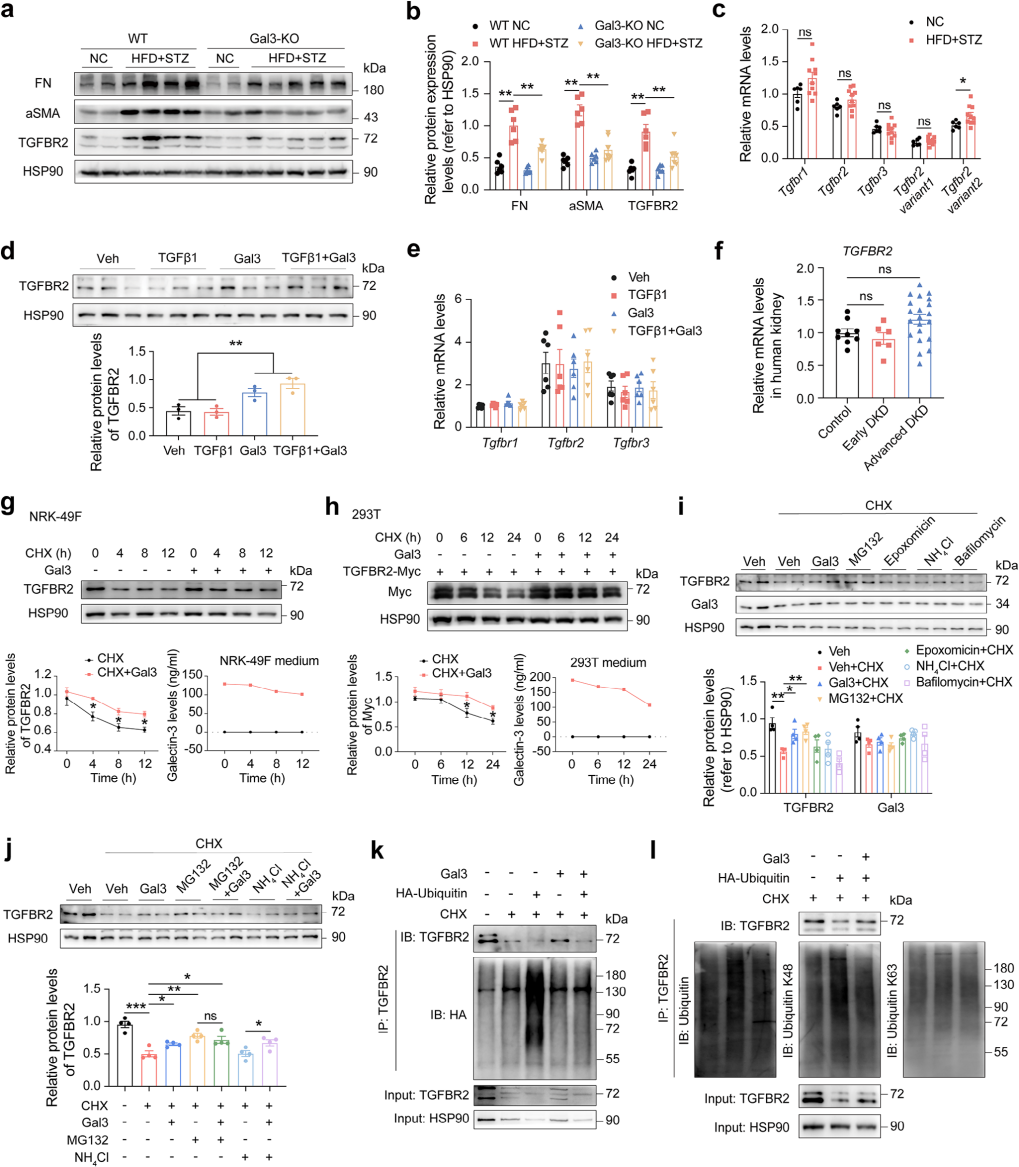
Gal3 inhibits the ubiquitin‐proteasome degradation pathway of TGFBR2. a) Protein levels of FN, aSMA and TGFBR2 in the kidney of WT and Gal3‐KO mice with or without DKD (n = 6 mice for NC‐fed WT, Gal3‐KO mice and DKD WT mice, n = 7 mice for DKD Gal3‐KO mice). b) statistical analysis of (a). c) mRNA levels of TGFβ1 receptors in WT mice with or without DKD (n = 6–10 mice per group). d) Protein levels of TGFBR2 in NRK‐49F fibroblasts after TGFβ1 and Gal3 treatments (n = 3 independent cell samples per group). e) mRNA levels of TGFβ1 receptors after TGFβ1 and Gal3 treatments (n = 6 independent cell samples per group). f) Human kidney mRNA levels of *TGFBR2* in control, early DKD and advanced DKD patients (from GSE142025 dataset,^[^ ^26^ ^]^ n = 9 patients for control, n = 6 patients for early DKD, n = 21 patients for advanced DKD). g) Protein levels of TGFBR2 after Gal3 and CHX treatments (n = 3 independent biological experiments) and Gal3 levels in cell medium of NRK‐49F fibroblasts. h) Protein levels of TGFBR2‐Myc after Gal3 and CHX treatments (n = 3 independent biological experiments) and Gal3 levels in cell medium of 293T cells. i) Protein levels of TGFBR2 and Gal3 in NRK‐49F fibroblasts after Gal3, proteasome inhibitor (MG132 and Epoxomicin) and lysosome inhibitor (NH4Cl and Bafilomycin) treatments (n = 4 independent cell samples per group). j) Protein levels of TGFBR2 in NRK‐49F fibroblasts after MG132 and NH4Cl treatments with or without Gal3 (n = 4 independent cell samples per group). k) Gal3 inhibited the ubiquitination of TGFBR2 in NRK‐49F fibroblasts treated with CHX. l) Gal3 inhibits K48‐linked TGFBR2 ubiquitination. Data were analyzed by two‐tailed Student's t test and presented as the mean ± SEM. * *p* < 0.05; ** *p* < 0.01; compared with indicated groups.

To investigate the regulatory effect of Gal3 on TGFBR2 expression, we examined TGFBR2 levels in the kidneys of DKD mice. Results showed that TGFBR2 protein levels were dramatically increased in the kidneys of DKD mice and decreased in Gal3‐KO mice, accompanied by changes in fibrosis‐related proteins (**Figure** **5**a,b). However, *Tgfbr2* mRNA levels remained stable between DKD and normal mice (Figure 5c). In renal fibroblasts, Gal3 treatment also elevated TGFBR2 protein levels without altering mRNA levels (Figure 5d,e). Transcriptome sequencing data (GSE142025)^[^ ^25^ ^]^ confirmed stable renal TGFBR2 mRNA levels in DKD and control patients (Figure 5f). These results indicated that Gal3 might interfere with TGFBR2 protein degradation.

Indeed, Gal3 inhibited TGFBR2 protein degradation in both renal fibroblasts and HEK‐293T cells (Figure 5g,h). To explore the underlying pathway, renal fibroblasts were treated with ubiquitin‐proteasome inhibitors (MG132 and Epoxomicin) and lysosomal inhibitors (NH_4_Cl and Bafilomycin). Results showed that Gal3 and MG132 significantly inhibited TGFBR2 protein degradation, while other inhibitors had no significant effects (Figure 5i). Co‐treatment with Gal3 and NH_4_Cl, but not MG132, enhanced TGFBR2 degradation inhibition (Figure 5j). These results indicated that Gal3 inhibited TGFBR2 protein degradation through the ubiquitin‐proteasome pathway. Furthermore, TGFBR2 ubiquitination analysis in renal fibroblasts and DKD mice kidneys confirmed Gal3's inhibitory effect on ubiquitination (Figure 5k; Figure S6a, Supporting Information).

To investigate the effect of Gal3 on TGFBR2 ubiquitin linkage patterns, TGFBR2 ubiquitination was assessed using total ubiquitin, K48‐linkage‐specific, and K63‐linkage‐specific antibodies after Gal3 treatment. The total ubiquitin and K48‐linkage‐specific antibodies showed similar patterns (Figure 5l). Ubiquitin K48R and K63R site mutation plasmids were generated for further validate. Consistent with previous results, the K48R mutation reduced TGFBR2 ubiquitination and diminished Gal3's inhibitory effect, while the K63R mutation had minimal impact on Gal3‐mediated inhibition (Figure S6a, Supporting Information). These findings suggested that Gal3 primarily inhibited K48‐linked ubiquitination of TGFBR2.

Overall, Gal3 inhibited TGFBR2 degradation through the ubiquitin‐proteasome pathway, promoting renal fibroblast activation and fibrosis during DKD.

### 2.6 Gal3 Binds to Pro‐TGFβ1 and Inhibits Its Degradation

**Figure 6:**
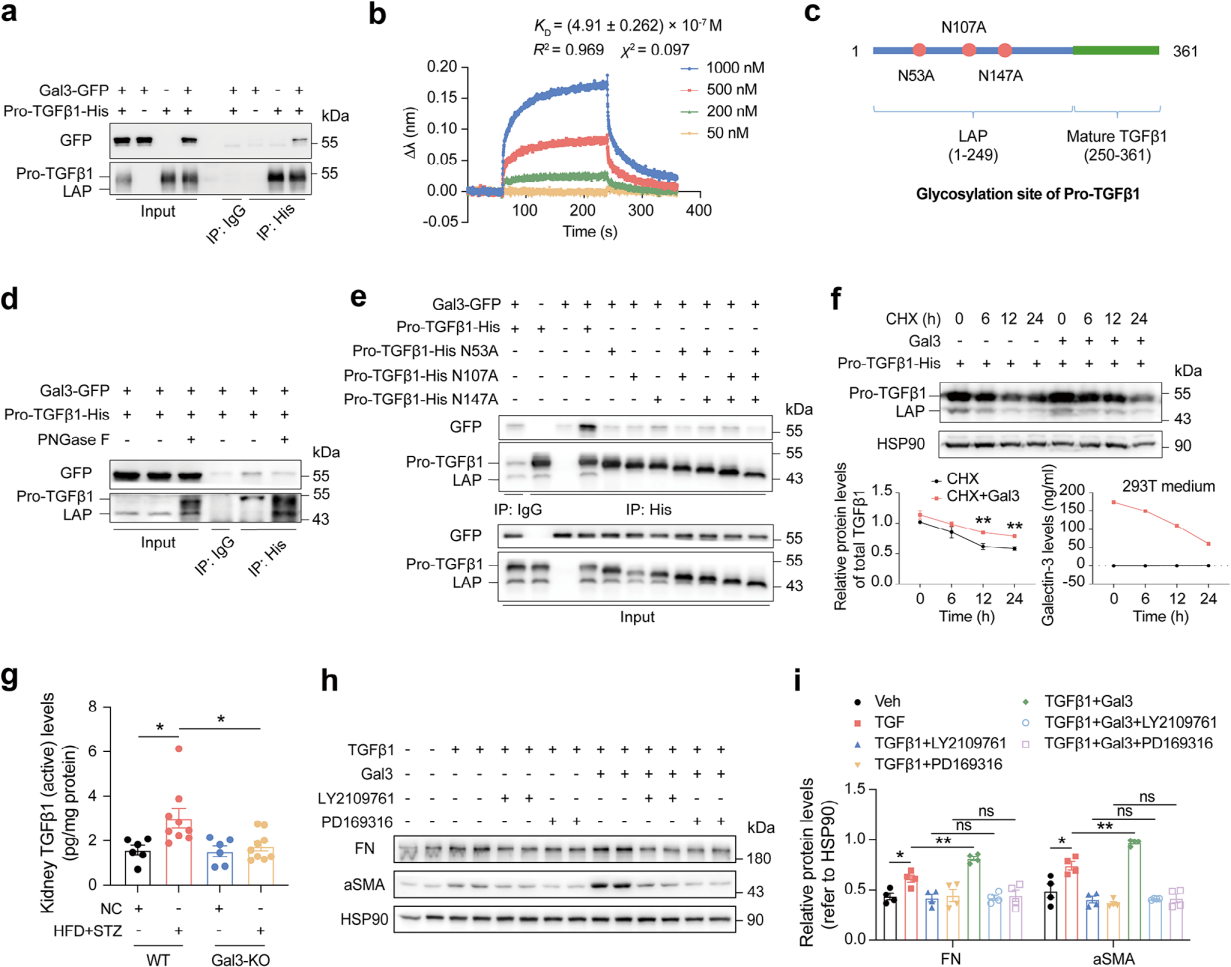
Gal3 binds with Pro‐TGFβ1 and inhibits its degradation. a) Co‐immunoprecipitation of Gal3‐GFP and Pro‐TGFβ1‐His in 293T cells. b) Gal3 and Pro‐TGFβ1 binding affinity assay using a BLI system. c) Structure diagram of Pro‐TGFβ1, red circle, glycosylation site. d) Co‐immunoprecipitation of Gal3‐GFP and Pro‐TGFβ1‐His in 293T cells after PNGase F treatment. e) Co‐immunoprecipitation of Gal3‐GFP and Pro‐TGFβ1‐His with glycosylation site mutation in 293T cells. f) Protein levels of Pro‐TGFβ1 and LAP after Gal3 and CHX treatments (n = 3 independent biological experiments) and Gal3 levels in cell medium of 293T cells. g) Mature TGFβ1 levels in the kidney of WT and Gal3‐KO mice with or without DKD (n = 6–9 mice per group). h) Protein levels of FN and aSMA in NRK‐49F fibroblasts after TGFBR2 inhibitor LY2109761 and TGFβ1 signaling inhibitor PD169316 treatments with or without Gal3 and TGFβ1 (n = 4 independent cell samples per group). i) Statistical analysis of (h). Data were analyzed by two‐tailed Student's t test and presented as the mean ± SEM. * *p* < 0.05; ** *p* < 0.01; compared with CHX group or indicated groups.

In the body, the majority of TGFβ1 exists as an inactive precursor form called Pro‐TGFβ1, which consists of the active form of TGFβ1 and the latency‐associated peptide (LAP) (**Figure** **6**c).^[^ ^31^ ^]^ Under certain conditions, such as treatment with proteases or integrins, TGFβ1 is released from Pro‐TGFβ1 and exerts its profibrotic effects.^[^ ^31^ ^]^

During our study, we serendipitously found that Gal3 could bind to Pro‐TGFβ1, as demonstrated by co‐immunoprecipitation (Figure 6a). Bio‐layer interferometry (BLI) assay confirmed the direct interaction between Gal3 and Pro‐TGFβ1, and revealed a high binding affinity, with an equilibrium dissociation constant (*K*_D_) of (4.91±0.262)×10^−7^ M (Figure 6b). The LAP component of Pro‐TGFβ1 contains three glycosylation sites (N53, N107 and N147) (Figure 6c). Treatment of Pro‐TGFβ1 with PNGase F to remove glycosylation, as well as point mutations at the glycosylation sites, both reduced its binding to Gal3. (Figure 6d,e). Among these, the N 53A point mutation had the most significant impact on the interaction. Additionally, co‐immunoprecipitation using truncated variants of Gal3 confirmed that Pro‐TGFβ1 binds to the CRD of Gal3 (Figure S6e, Supporting Information).

Interestingly, we found that Gal3 also inhibits the degradation of Pro‐TGFβ1 (Figure 6f). In DKD mice, the renal content of TGFβ1 was significantly increased, whereas it was significantly reduced following Gal3 ablation (Figure 6g). Since TGFβ1 is primarily released from extracellular Pro‐TGFβ1, we hypothesized that Gal3 may also increase renal TGFβ1 levels in DKD by slowing Pro‐TGFβ1 degradation, thus contributing to renal fibrosis.

To confirm whether Gal3 promotes renal fibrosis mainly through the regulation of the TGFβ1 signaling pathway, we treated renal fibroblasts with Gal3 and TGFβ1, along with either the TGFBR2 inhibitor (LY2109761) or the TGFβ1 signaling pathway inhibitor (PD169316). The results showed that both inhibitors significantly suppressed TGFβ1‐induced fibroblast activation. Furthermore, Gal3 addition did not enhance the inhibitory effects of either inhibitor (Figure 6h,i), suggesting that Gal3 promotes renal fibroblast activation and fibrosis mainly by enhancing the activation of TGFβ1 signaling pathway.

### 2.7 Pharmacological Inhibition of Gal3 Ameliorates Kidney Fibrosis in DKD Mice

**Figure 7:**
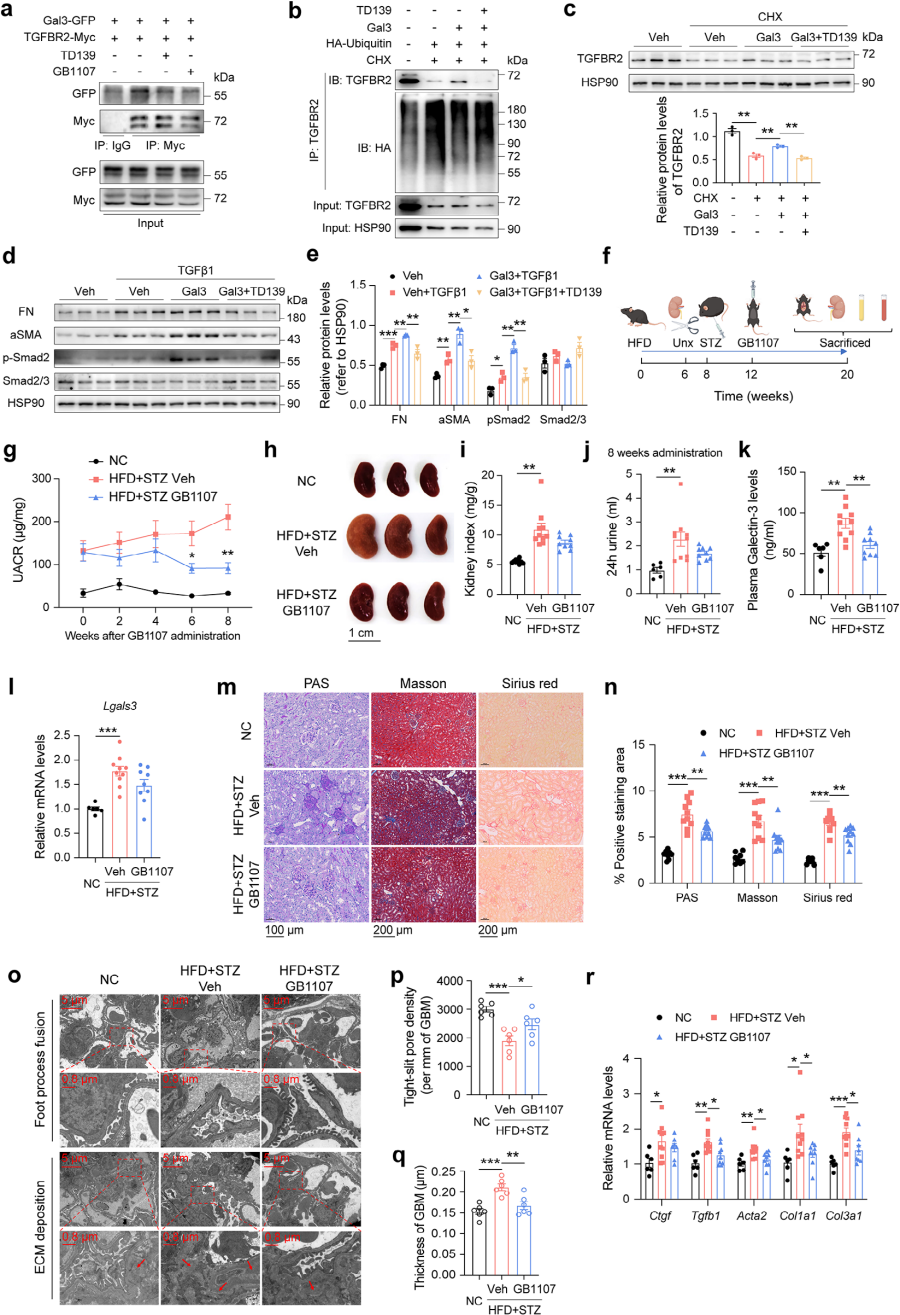
Pharmacological inhibition of Gal3 ameliorates kidney fibrosis in DKD mice. a) Co‐immunoprecipitation of Gal3‐GFP and TGFβ1‐Myc in 293T cells after Gal3 inhibitors TD139 and GB1107 treatments. b) Gal3 inhibitor TD139 restored the ubiquitination of TGFBR2 in NRK‐49F fibroblasts after Gal3 and CHX treatments. c) Protein levels of TGFBR2 in NRK‐49F fibroblasts after Gal3, TD139 and CHX treatments (n = 3 independent cell samples per group). d) Protein levels of FN, aSMA, p‐Smad2 and Smad2/3 in NRK‐49F fibroblasts after Gal3, TD139 and TGFβ1 treatments (n = 3 independent cell samples per group). e) Statistical analysis of (d). f) Schematic diagram of experiment. g**–**i) UACR (g), representative kidney images (h) and kidney index (left kidney weight / body weight) (i) of DKD mice after GB1107 treatment. j) Volume of 24 hours urine in DKD mice after 8 weeks GB1107 treatment. k, l) Plasma (k) and kidney (l) Gal3 levels in DKD mice after GB1107 treatment. m, n) PAS, Masson and Sirius red staining of kidney in NC, DKD mice with vehicle or GB1107 treatments, representative images (m) and statistical analysis (n) (scale bar 100 µm for PAS and 200 µm for Masson and Sirius red). o–q) TEM images (o) and statistical analysis of tight‐slit pore density (p) and thickness of GBM (q) in NC, DKD mice with vehicle or GB1107 treatments (scale bar 5 µm or 0.8 µm as indicated, red arrow, ECM deposition). r) Kidney mRNA expression levels of fibrosis related genes in NC, DKD mice with vehicle or GB1107 treatments. n = 6–10 mice per group in g‐r. Data were analyzed by two‐tailed Student's t test and presented as the mean ± SEM. *p < 0.05; **p < 0.01; ***p < 0.001; compared with indicated groups.

To investigate the potential of Gal3 as a therapeutic target for renal fibrosis and DKD, we treated renal fibroblasts and DKD mice with Gal3 inhibitors TD139 and/or GB1107. Our results showed that both inhibitors significantly reduced the binding of Gal3 to TGFBR2 (**Figure** **7**a) and Pro‐TGFβ1 (Supplementary Figure S6a). TD139 also restored the ubiquitination and degradation of TGFBR2 (Figure 7b,c) and the degradation of Pro‐TGFβ1 (Figure S6b, Supporting Information), which were inhibited by Gal3. Moreover, the TGFβ1 signaling enhancing effect of Gal3 was blocked by TD139 (Figure 7d,e).

Furthermore, we administrated GB1107 to DKD mice via gavage for 8 weeks to examine its in vivo therapeutic effects (Figure 7f). Similar to Gal3‐KO mice, GB1107 administration had no effect on body weight or FBG (Figure S6c,d, Supporting Information), but it significantly improved the kidney function of DKD mice (Figure 7g–i). The volume of 24‐hour urine, as well as the amounts of food and water intake in DKD mice, were decreased after GB1107 treatment (Figure 7j; Figure S6e, Supporting Information), suggesting an improvement in diabetic symptoms in DKD mice. Intriguingly, GB1107 treatment led to a decrease in plasma and kidney Gal3 levels in DKD mice (Figure 7k,l).

Kidney histology and TEM analysis revealed a remarkable amelioration of renal fibrosis, inflammation and microstructural lesions in DKD mice after GB1107 administration (Figure 7m–q; Figure S6f, Supporting Information). Meanwhile, the expressions of genes related to fibrosis and inflammation were downregulated, and the TGFβ1 signaling pathway and TGFBR2 protein levels were also inhibited in the kidneys of DKD mice treated with GB1107 (Figure 7r; Figure S6g–k, Supporting Information). These findings suggest that the pharmacological inhibition of the excessively expressed Gal3 in DKD represents a promising therapeutic strategy for renal fibrosis and DKD.

## 3 Discussion

DKD is one of the most common complications of diabetes. Renal fibrosis is a major consequence of DKD patients, yet effective treatments remain limited.^[^ ^32^ ^]^ Our previous studies showed that Gal3 is associated with insulin resistance and islet β‐cell dysfunction in obesity and diabetes.^[^ ^28^, ^29^ ^]^ Gal3 is also implicated in various inflammatory and fibrotic diseases; however, its direct involvement in the pathogenesis of renal fibrosis and DKD has remained elusive. In this study, we confirmed the imperative role of macrophage‐derived Gal3 in renal fibrosis and DKD. Inhibiting the abnormally expression of Gal3 significantly alleviated renal fibrosis in mice. We also discovered a novel mechanism: Gal3 directly binds to TGFBR2 on renal fibroblasts and Pro‐TGFβ1 in the kidney. This binding inhibits the ubiquitin‐proteasome‐mediated degradation of TGFBR2 and increases TGFβ1 levels, thereby enhancing the profibrotic effects of the TGFβ1 signaling pathway and promoting the progression of renal fibrosis and DKD.

Our results showed that Gal3 levels in the blood and kidneys of patients increased gradually with the progression of diabetes or DKD and were significantly correlated with renal fibrosis (Figure 1), which is consistent with previous studies.^[^ ^12^ ^]^ Gal3 has been reported to function as an advanced glycation end‐products (AGEs) receptor (RAGE), facilitating the clearance of excessive AGEs in glomeruli during diabetic glomerulopathy. In mice, ablation of Gal3 leads to AGEs accumulation and accelerates diabetic glomerulopathy.^[^ ^33^ ^]^ Conversely, subsequent studies have shown that Gal3 knockout promotes diabetic retinal angiogenesis and inhibits the development of diabetic retinopathy by improving blood‐retinal barrier dysfunction in diabetic patients.^[^ ^34^, ^35^ ^]^ These studies indicate the complex role of Gal3 in diabetic complications. Besides DKD, Gal3 also participates in the development of various acute and chronic kidney diseases.^[^ ^17^, ^18^, ^19^ ^]^ Given the dual functions of RAGE (mediating extracellular AGEs clearance and facilitating their entry into cells, causing stress) and the limited impact of AGEs on CKD, Gal3 may exert additional effects on DKD and CKD. In this study, we found that Gal3 binds to TGFBR2, potentiating the effect of TGFβ1 and promoting renal fibrosis, consistent with reports of Gal3‐induced fibrosis in other organs.^[^ ^15^ ^]^

Gal3 is widely expressed in tissues and cells, especially in activated macrophages.^[^ ^28^, ^36^ ^]^ During DKD, immune disorder and inflammation are increased, with macrophages showing an elevation exceeding 50%.^[^ ^37^, ^38^, ^39^, ^40^, ^41^ ^]^ Conditional depletion of macrophages in diabetic mice substantially mitigated kidney injury, and chimeric experiments indicated that most macrophages originated from the bone marrow.^[^ ^40^ ^]^ Nevertheless, the mechanisms by which macrophages contribute to DKD remain elusive. This study demonstrates that Gal3, primarily secreted by activated macrophages, is crucial for renal fibrosis in DKD, highlighting its significant role in macrophage‐mediated kidney injury. Further research is required to clarify how Gal3 regulates immune dysfunction in diabetic kidneys. Notably, Gal3 has been shown to modulate inflammatory signaling in various diseases, such as atherosclerosis, pulmonary disorders, and nonalcoholic steatohepatitis.^[^ ^42^, ^43^ ^]^ In our study, Gal3 knockout also alleviated kidney inflammation in DKD mice (Figures S2d, g, S3d, and S6g, Supporting Information), suggesting a potential association between Gal3 and inflammation in DKD progression.

The activation of fibroblasts to myofibroblasts is a pivotal process in organ fibrosis and scar formation.^[^ ^44^ ^]^ Henderson et al. demonstrated that Gal3 initiates the activation of liver fibroblasts and promotes CCL4‐induced liver fibrosis. Although Gal3 ablation did not affect *Tgfb1* expression in the liver, it inhibited the expression of downstream markers, such as aSMA and collagens, suggesting that Gal3 may regulate TGFβ1 signal transduction.^[^ ^45^ ^]^ In models of TGFβ1‐ and bleomycin‐induced pulmonary fibrosis, Gal3 knockout significantly reduced TGFβ1‐induced epithelial‐mesenchymal transition of alveolar epithelial cells and fibroblast activation through inhibiting the β‐catenin signaling pathway.^[^ ^46^ ^]^ These studies highlight the significant role of Gal3 in fibroblast activation and fibrosis, suggesting that Gal3 alone can trigger fibroblast activation. Notably, our study showed that Gal3 treatment alone failed to activate renal fibroblasts, yet it potentiated the activating effect of TGFβ1 on these cells. This discrepancy may be attributed to differences in Gal3 dosage. We found that Gal3 plasma concentrations were low in healthy controls and typically ranged from 30–200 ng mL^−1^ in DKD patients and mice, indicating that the physiological and pathological levels of Gal3 under DKD conditions peak at approximately 200 ng/ml. At this concentration, the capacity of Gal3 to activate fibroblasts on its own appears to be limited.

We found that Gal3 deletion significantly mitigated renal fibrosis in DKD mice and concurrently reduced renal *Tgfb1* expression (Figure 2). To confirm whether Gal3 directly regulates the expression of *Tgfb1*, we overexpressed Gal3 in various types of kidney cells. The results showed that there was no significant increase in *Tgfb1* levels (Figure 4a; Figure S5a–d, Supporting Information). Meanwhile, treating fibroblasts with Gal3 also had no significant impact on *Tgfb1* expression (Figure S5h, Supporting Information). This suggests that the reduced renal *Tgfb1* expression in Gal3‐KO DKD mice may be a secondary effect of the improved renal inflammatory and fibrotic environment. In addition, we found that Gal3 could directly bind to Pro‐TGFβ1, inhibiting its degradation and potentially increasing TGFβ1 levels in DKD kidneys. However, considering the activation modes of TGFβ1,^[^ ^47^ ^]^ we speculate that Gal3 may also participate in the activation of Pro‐TGFβ1 mediated by thrombospondin‐1 or integrins. Notably, a recently published study has shown that Gal3 can induce integrin‐mediated TGFβ1 activation in human lung fibroblasts.^[^ ^48^ ^]^ Further studies are needed to clarify the specific contributions of these mechanisms to renal fibrosis and DKD.

Interestingly, our results showed that TGFβ1 suppressed Gal3 expression in fibroblasts (Figure 4c; Figure S5e, f, Supporting Information), whereas overexpression of Gal3 in fibroblasts inhibited the profibrotic effects of TGFβ1 (Figure 4e,f), which contrasts with the function of extracellular Gal3. Intracellular and extracellular Gal3 have distinct roles: intracellular Gal3 is involved in cell growth, proliferation, and anti‐apoptosis,^[^ ^49^ ^]^ while extracellular Gal3 interacts with membrane proteins to mediate signal transduction.^[^ ^50^ ^]^ Given that TGFβ1 is a potent profibrotic cytokine with inhibitory effects on cell growth and proliferation,^[^ ^51^, ^52^ ^]^ this may account for the decrease in intracellular Gal3 levels. Overexpression of Gal3 in fibroblasts increased its intracellular levels, potentially promoting cell growth and proliferation, which could counteract the effects of TGFβ1. Thus, elevated intracellular and extracellular Gal3 levels have opposing effects on TGFβ1 signaling.

Our previous findings demonstrated that Gal3 impairs islet β‐cell function and insulin secretion in obese and diabetic mice.^[^ ^24^ ^]^ However, there were no significant changes in the body weight or blood glucose in DKD mice after Gal3 ablation or Gal3 inhibitor administration (Figures S2a, b, and S6c–e, Supporting Information). This divergence maybe due to differences in diabetic mice models. The DKD mice used in this study had a longer modeling period and severer islet damage compared to the obese and diabetic mice used previously. Consequently, the impact of Gal3 on islet β‐cell dysfunction might have been limited. Besides its effects on tubular interstitial fibrosis, we found that Gal3 inhibition significantly improved glomerulosclerosis and podocyte injury in DKD mice (Figure 7o–q). These results suggest that Gal3 may also participate in the regulation of glomerular interstitial cell and podocyte injury, although the underlying mechanisms require further investigation.

In conclusion, this study elucidated the critical role of Gal3 in renal fibrosis and DKD. Extracellular Gal3, released mainly by macrophages, contributes to the progression of renal fibrosis. Genetic or pharmacological inhibition of the abnormally increased Gal3 levels notably improves renal function and attenuates fibrosis in DKD mice. Mechanistically, macrophage‐secreted Gal3 directly binds to TGFBR2 in renal fibroblasts, suppressing its ubiquitination and degradation. Additionally, Gal3 interacts with Pro‐TGFβ1, directly or indirectly increasing TGFβ1 levels in DKD kidneys. The augmented TGFBR2 and TGFβ1 levels in diabetic kidneys potentiate the TGFβ1 signaling pathway, thereby facilitating renal fibrosis development. These findings identify a potential therapeutic target and provide new perspectives for the treatment of renal fibrosis and DKD.

## 4 Experimental Section

### Study Approval

All animal experiments were conducted according to the approved experimental protocol by the Animal Experiment Ethics Committee of the Chinese Academy of Medical Sciences, and all procedures were conducted in accordance with the Animal Research: Reporting of In Vivo Experiments (ARRIVE) guidelines. The use of clinical samples in this study has been reviewed and approved by the Research Ethics Committee of the Affiliated Hospital of Xuzhou Medical University (Ethical approval No. XYFY2019‐KL222‐01) and the Research Ethics Committee of Beijing Friendship Hospital Affiliated to Capital Medical University (Ethical approval No. 2024‐P2‐239‐01).

### Statistical Analysis

All data were presented as mean ± SEM and detailed information was indicated in the figure legends. Images were processed and analyzed by Zeiss Zen Blue software and Image J software; numerical data were analyzed by Prism 9 software. Pearson correlation coefficient *r* was used to represent the correlations between the two groups, unpaired two‐sided student's *t*‐test was used to represent the differences between the two groups and *P* ≤ 0.05 was regarded as statistically significant.

The full and detailed methods were described in the Supporting Information.

## Conflict of Interest

The authors declare no conflict of interest.

## Author Contributions

Y.C. and Q.J. contributed equally to this work. Y.C. performed most of the experiments and analyzed the data; Q.J. generated macrophage Gal3 specific‐knockout mice; Q.J., X.X., J.C. assisted with animal experiments; Q.J., X.X., assisted with collecting tissues; Q.Z., C.M., X.L. assisted with plasmid construction and biochemistry experiments; Q.L., L.X., L.D. provided the human serum; H.H., Q.Z. provided the human kidney sections; J.G. helped animal modeling; P.L. conceived the project and directed the research. Y.C. and P.L. wrote the manuscript. All authors contributed to the design or revision of the study.

## Supporting information



Supporting Information

## Data Availability

The clinical kidney RNA‐seq data was acquired from Gene Expression Omnibus (GEO) dataset under the identifier GSE142025. Clinical samples were collected from the Affiliated Hospital of Xuzhou Medical University (ethics approval No. XYFY2019‐KL222‐01) and Beijing Friendship Hospital, affiliated with Capital Medical University (ethics approval No. 2024‐P2‐239‐01), China. The data collected for this study will be made available as deidentified participant data to researchers who propose to use it for individual patient data meta‐analysis. Data will be shared upon approval of the proposal by the corresponding author.
