# Supplementary material for: Macrophage Derived Galectin‐3 Promotes Renal Fibrosis and Diabetic Kidney Disease by Enhancing TGFβ1 Signaling
Source: Adv Sci (Weinh). 2025 Aug 13;12(35):e04032. doi: 10.1002/advs.202504032 (PMC12462920; doi:10.1002/advs.202504032)
Supplement: Supplementary file 1 — Supporting Information [file ADVS-12-e04032-s001.docx]

**Supplementary Methods:**

**Clinical samples**

Serum samples of normal individuals, diabetic patients and DKD patients were obtained from the Affiliated Hospital of Xuzhou Medical University. The use of plasma samples has been approved by the Research Ethics Committee of the Affiliated Hospital of Xuzhou Medical University. In this study, we included 15 normal individuals, 29 diabetic patients without kidney disease and 28 DKD patients (Donors’ information can be found in Supplementary Table 1).

Renal biopsy samples of DKD patients and control patients used in this study were obtained from Beijing Friendship Hospital, Capital Medical University. The use of renal biopsy samples has been approved by the Research Ethics Committee of Beijing Friendship Hospital, Capital Medical University. In this study, we included 6 DKD patients and 3 control patients (Donors’ information can be found in Supplementary Table 2).

The gene expression data in the kidneys of DKD patients used in this study were sourced from the NCBI GEO database GSE142025 dataset.^1^ This dataset includes 9 normal individuals, 6 early-stage DKD patients and 21 late-stage DKD patients.

**Animal experiments**

All animals were kept in the SPF animal house with regular dark / light circle (12 hours /12 hours), constant temperature (25℃) and humidity (50-60% humidity), adequate food and water. Special conditions were characterized in the experiments.

1. *db/db* mice induced DKD model

4 weeks old female *db/db* mice and their control mice were purchased from Beijing Huafukang Biotechnology, China. At 6 weeks of age, *db/db* mice were anesthetized with tribromoethanol and the right kidneys were removed to accelerate the development of nephropathy. *db/db* mice were sacrificed at 8 weeks of age, 16 weeks of age, 24 weeks of age and 36 weeks of age, samples were collected to detect the development of kidney disease and renal fibrosis, and the expression of Gal3 in mice. Sample collection: Mouse kidney, plasma and urine, etc. were collected for follow-up experiments.

2. High-fat diet (HFD) feeding with streptozotocin (STZ) intraperitoneal injection induced mice DKD model

In this study, we performed two kinds of HFD+STZ modeling methods to induce mice DKD.

A. Mice were fed with HFD (D12492, Research Diets, USA) for 8 weeks from 8 weeks of age. After fasting overnight, mice were treated with STZ (S0130, Sigma-Aldrich, USA) through intraperitoneal injection for consecutive 3 days (50 mg STZ / kg body weight, STZ was dissolved in citrate buffer, pH 4.0-4.5). Blood glucose was detected 7 days after the last injection, fasting blood glucose higher than 11.1 mM or random blood glucose higher than 16.7 mM was considered a successful diabetic model.^2^ Then, HFD feeding was continued for another 12 weeks before the right kidneys of the mice were removed. Mice were fed with HFD for another 20 weeks (a total 40 weeks HFD feeding) and sacrificed.

B. Mice were fed with HFD for 6 weeks from 6 weeks of age. The right kidneys of the mice were removed and the STZ administration was performed 2 weeks after nephrectomy. Mice were fed with HFD for another 8-16 weeks (a total 16-24 weeks HFD feeding) and sacrificed.

Gal3 whole body knockout (Gal3-KO) mice and wild-type (WT) control mice were a gift from Professor Jerrold M. Olefsky, University of California, San Diego, USA. Lyz2-icre mice were purchased from GemPharmatech, China. And ZK-003CKO (Gal3 f/f) mice were purchased from BIOCYTOGEN, China. Lyz2-icre mice were crossed with Gal3 f/f mice to generate macrophage Gal3-specific knockout (Gal3 MKO) mice as previously described.^3^

Sample collection: Mouse kidney, plasma and urine, etc. were collected for follow-up experiments.

Note: Since the modeling method B takes shorter time than the modeling method A and the degree of kidney injury and renal fibrosis is also severer in method B, we used method B for mice DKD modeling in the majority of studies as indicated in the manuscript.

3. 5/6 nephrectomy induced mice CKD model

8 weeks old WT and Gal3-KO mice were anesthetized with tribromoethanol, and the left kidneys were nephrectomized by 2/3 (the upper blood vessels and the lower ureter were dissected out, and the upper and lower 1/3 of the kidney were removed respectively and ligated to prevent bleeding). After 2 weeks recovery, the right kidneys of the mice were removed. Mice were sacrificed 16 weeks later after 5/6 nephrectomy. Sample collection: Mouse kidney, plasma and urine were collected for follow-up experiments.

4. Gal3 inhibitor GB1107 administration in DKD mice

4 weeks old C57BL6/J wildtype male mice were purchased from Beijing Vital River Laboratory Animal Technology, China. And the mice were induced into DKD according to the modeling method B mentioned above. After modeling for 4 weeks, the DKD mice were equally divided into two groups according to body weight, FBG and UACR, and treated with Gal3 inhibitor GB1107 (10 mg/kg/day; HY114409, MedChemExpress, USA) or the same amount of control solvent (distilled water containing 0.1% Tween 80) by gavage respectively. Food intake and water intake were monitored, and blood and urine of the mice were taken every 2 weeks during GB1107 administration. Mice were sacrificed 8 weeks after GB1107 treatment. Sample collection: Mouse kidney, plasma and urine, etc. were collected for follow-up experiments.

**Cell experiments**

All cells were cultured in an incubator with constant temperature (37℃) and 5% CO_2_. RAW 264.7, NIH/3T3 and HEK-293T cell lines were gifted from Professor Bing Cui, Institute of Materia Medica, Chinese Academy of Medical Sciences, China. MPC-5 cell line was gifted from Professor Xiaoxing Yin, Xuzhou Medical University, China. M-1 cell line (1101MOU-PUMC000223) was purchased from National Experimental Cell Resource Sharing Platform, China. HK-2 cell line was gifted from Professor Xuemei Li, Peking Union Medical College Hospital, Chinese Academy of Medical Sciences, China. NRK-49F cell line (CL0349) was purchased from Hunan Fenghui Biotechnology, China. RAW 264.7, NIH/3T3, HEK-293T, MPC-5 and NRK-49F were cultured in DMEM-HG medium (12800-017, Gibco, USA) with 10% fetal bovine serum (16000044, Gibco, USA) and 100 U/mL penicillin and streptomycin (FG101-01, TransGen Biotech, China). M-1 and HK-2 were cultured in DMEM/F12 medium (CM10090, M&C Gene Technology, China) with 10% fetal bovine serum and 100 U/mL penicillin and streptomycin.

1. Gal3 secretion experiment

RAW 264.7, NIH/3T3, MPC-5, M-1 and HK-2 cells were seeded in 6-well plates. Cells with 70% confluency were starved with DMEM-LG medium (11885-084, Gibco, USA) with 1% fetal bovine serum and 100 U/mL penicillin and streptomycin for 12 hours, and then treated with high-glucose (HG) (30 mM, 10010592, Sinopharm Chemical Reagent, China), LPS (20 ng/mL, L4391, Sigma-Aldrich, USA) or TGFβ1 (5 ng/mL or 10 ng/mL, 80116-RNAH, Sino Biological, China) protein for 24 hours, or palmitic acid (PA) (200 μM, P0002, TCI, Japan) for 8 hours. The control group was treated with the same amount of PBS buffer as a control. The medium and cells were harvest. Gal3 secretion in cells (ng/mg) = Total amount of Gal3 in cell medium (Gal3 concentration in medium × volume) / Total amount of cells (total protein concentration in cell lysate × volume)

2. Cell transfection

RAW 264.7, NIH/3T3, MPC-5, M-1 and HK-2 cells were seeded in 6-well plates. Cells with 70% confluency were transfected with Gal3-GFP (mouse) plasmid or Gal3-Myc (human) plasmid or control vehicle plasmid (1 μg/well, homemade) followed the instruction of Lipo8000^TM^ transfection kit (C0533, Beyotime Biotechnology, China). The Medium was refreshed after 6 hours of transfection, and the cells were harvested 36 hours after transfection for RNA extraction and subsequent experiments. 293T cells were transfected with Pro-TGFβ1-His (MG50698-NH, Sino Biological, China) plasmid or TGFBR1-Myc (MG50238-CM, Sino Biological, China) plasmid or TGFBR2-Myc (MG57076-CM, Sino Biological, China) plasmid or HA-Ubiquitin plasmid (homemade) with the instructions describe above.

3. Gal3 and TGFβ1 treatment

NRK-49F and NIH/3T3 cells were seeded in 6-well plates. Cells with 70% confluency were starved with DMEM-HG medium with 1% fetal bovine serum and 100 U/mL penicillin and streptomycin for 12 hours, and then treated with 200 ng/mL or indicated concentrations of Gal3 (1197-GA-050, R&D Systems, USA) and 5 ng/mL TGFβ1 (80116-RNAH, Sino Biological, China) for 24 hours before harvest.

4. Cycloheximide (CHX) treatment

NRK-49F and HEK-293T cells were treated with 20 μg/ml CHX (S7418, Selleck, USA) in indicated conditions.

5. Proteasome and lysosome inhibitors treatment

NRK-49F cells were treated with proteasome inhibitors 10 μM MG132 (HY-13259, MedChemExpress, USA) and 2 μM Epoxomicin (HY-13821, MedChemExpress, USA), and lysosome inhibitors 5 mM NH_4_Cl (213330, Sigma-Aldrich, USA) and 50 nM Bafilomycin A1 (HY-100558, MedChemExpress, USA) for 24 hours before harvest.

6. Deglycosylation treatment

Protein lysate of cells was treated with PNGase F (20407ES01, Yeasen Biotech, China) following the instructions of the kit.

7. TGFβ1 signaling pathway inhibitors treatment

NRK-49F cells were treated with 5 μM TGFBR2 inhibitor LY2109761 (HY-12075, MedChemExpress, USA) and 10 μM TGFβ1 signaling inhibitor PD169316 (HY-10578, MedChemExpress, USA) for 24 hours before harvest.

8. Gal3 inhibitors treatment

NRK-49F and HEK-293T cells were treated with 1 μM Gal3 inhibitors TD139 (S0471, Selleck, USA) and GB1107 (HY114409, MedChemExpress, USA) for 24 hours before harvest.

**Kidney histology analysis**

The kidneys were dissected into two halves from longitudinal direction. Half of the kidneys were fixed in 4% paraformaldehyde for 48 hours and embedded in the paraffin as described before.^4^ 4 μm thickness renal tissue sections were used for further studies. Periodic acid Schiff (PAS) staining (G1008, Servicebio, China) was performed to assess the glycogen deposition, and Masson and Sirius red stainings (G1006; G1018, Servicebio, China) were conducted to investigate the collagen deposition of kidney followed the manufacture instructions. Hematoxylin-eosin (HE) staining (G1076, Servicebio, China) was performed to observe the general morphology of kidney tissue.

Immunohistochemistry was performed to examine the expressions of Gal3, F4/80, aSMA and FN in mice kidney. After deparaffinized in xylene and graded alcohol, the sections received endogenous peroxidase activity elimination and pepsin antigen retrieval. Then blocked with 3% bovine serum albumin (BSA) (BAH66-0050, Equitech-Bio, USA) for 1h at room temperature followed with primary antibodies incubation at 4℃ overnight. The primary antibodies against Gal3 (1:200), F4/80 (1:10000), aSMA (1:200) and FN (1:200) were obtained from Abcam (ab2785, UK), Proteintech (29414-1-AP, USA), Proteintech (14395-1-AP, USA) and Cell Signaling Technology (26836S, USA), respectively. Images were randomly taken under an automatic multispectral pathological section scanning analysis imaging system (Vectra 3, Perkin Elmer, USA) from random fields.

For quantification of positive staining area, we followed the protocol described before. briefly, after sections were stained with different reagents or antibodies. The images with positive staining areas were analyzed by Image J 2.1 software. Firstly, open a picture in the software and change the image type into RGB stack. Adjust the pulley below to the right and then chose the proper threshold until the positive area been shown (remember the threshold and apply to all images with the same threshold). Analyze the percentage of positive staining area (% Area).

**Immunofluorescence**

① NRK-49F cells were seeded in the 12 well plate with round glass coverslips and treated with Gal3 or TD139 for 12 hours. After fixed with methanol at -20℃ for 15 min and blocked with 2% BSA for 1h at room temperature, cells were incubated with primary antibodies against Gal3 (1:200, ab2785, Abcam, UK), TGFBR2 (1:200, 66636-1-Ig, Proteintech, USA), AQP1 (1:200, 20333-1-AP, Proteintech, USA), Podocalyxin (1:200, MAB1556, R&D Systems, USA), F4/80 (1:200, 29414-1-AP, Proteintech, USA) and aSMA (1:200, 14395-1-AP, Proteintech, USA) at 4℃ overnight. ② Human kidney specimen sections (4 μm) after deparaffinised were eliminated endogenous peroxidase with H_2_O_2_ and blocked with 2% BSA for 1h at room temperature, then incubated with primary antibodies against Gal3 (1:200) and TGFBR2 (1:200) at 4℃ overnight.

Then the coverslips with cells or kidney sections were washed by PBS and incubated with an anti-rabbit or anti-mouse secondary antibody conjugated with Alexa Fluor 488 (1:200, A11029 or A11034, Invitrogen, USA) or Alexa Fluor 546 (1:200, A10040 or A10036, Invitrogen, USA) at 37℃ for 1h. Cell membrane of NRK-49F cells were stained with phalloidin (1:200, A12381, Invitrogen, USA). Cell nuclei of kidney sections were stained with DAPI (P36935, Invitrogen, USA) for 2-5min at room temperature. Images were randomly taken using a Zeiss LSM 880 microscope. Pearson’s correlation coefficient was calculated using Image J software.

**Transmission electron microscopy**

Kidney tissues were fixed in 2.5% glutaraldehyde dissolved in 0.1 M phosphate buffer and processed for routine electron microscopy as described before.^5^ Images were acquired using a Hitachi Transmission Electron Microscope system. Image J 2.1 software was used for all quantifications.

**Urine albumin and creatinine ratio (UACR) and blood urea nitrogen (BUN)** **detection**

For UACR detection, the supernatant of mouse urine was used for the experiment. Urine was diluted 200-1000 times for albumin detection, following the instruction of mouse urine albumin ELISA kit (E99-134, Bethyl, USA). Dilute the urine 5-50 times to detect the creatinine levels, following the instruction of creatinine assay kit (C011-2-1, Nanjing Jiancheng Bioengineering Institute, China). UACR (μg/mg) = Urinary albumin concentration / Urinary creatinine concentration.

For BUN detection, plasma samples of mice were diluted 5-10 time and following the instruction of BUN assay kit (C013-2-1, Nanjing Jiancheng Bioengineering Institute, China).

**Gal3 and TGFβ1 levels detection**

For blood Gal3 detection, human serum samples were diluted 10 times and following the instruction of Human Galectin-3 ELISA kit (DGAL30, R&D Systems, USA). Plasma samples of mice were diluted 400-800 times and following the instruction of Mouse Galectin-3 ELISA kit (SK00199-03, Aviscera Bioscience, USA).

For cell medium Gal3 detection, cell medium samples were diluted 100 times and following the instructions described above.

For mouse kidney TGFβ1 detection, protein lysis of kidney was used and following the instruction of Mouse TGFβ1 ELISA kit (DB100C, R&D Systems, USA), the final concentrations of kidney TGFβ1 were calibrated with total protein concentration.

**Western blotting and Co-immunoprecipitation (Co-IP)**

Cells and mice kidneys were collected to investigate the relative proteins expression levels. Cells and kidney tissue were homogenized and lysed in Western & IP lysis buffer (P0013, Beyotime Biotechnology, China) containing 1% protease (HY-K0010, MedChemExpress, USA) and phosphatase inhibitor (HY-K0021/22, MedChemExpress, USA). The bicinchoninic acid (BCA) protein assay (PA115-02, TIANGEN Biotech, China) was performed to determine the protein concentration according to the manufacture instructions. The protein samples were separated with a SDS page and then transferred to PVDF membrane (IPVH00010, Millipore, USA). After incubated with primary antibodies against Gal3 (1:1000, ab2785, Abcam, UK), FN (1:1000, 26836S, Cell Signaling Technology, USA), aSMA (1:1000, 14395-1-AP, Proteintech, USA), HSP90 (1:2000, 60318-1-Ig, Proteintech, USA), JNK (1:1000, A5005, Selleck, USA), p-JNK (1:1000, 4668S, Cell Signaling Technology, USA), Smad2/3 (1:1000, 5678S, Cell Signaling Technology, USA), p-Smad2 (1:1000, 3108S, Cell Signaling Technology, USA), GFP (1:1000, 598, MBL, Japan), Myc (1:1000, 2276S, Cell Signaling Technology, USA), TGFBR2 (1:1000, 66636-1-Ig, Proteintech, USA), HA (1:1000, 561, MBL, Japan), Ubiquitin (1:1000, 58395S, Cell Signaling Technology, USA), Ubiquitin K48- (1:1000, F0527, Selleck, USA), Ubiquitin K63- (1:1000, F0528, Selleck, USA), Pro-TGFβ1 (1:1000, 21898-1-AP, Proteintech, USA) and E-Ca (1:1000, 20874-1-AP, Proteintech, USA) overnight at 4℃, membranes were washed by PBST and then incubated with secondary antibodies (1:10000, HRP-conjugated mouse anti-rabbit, BE0101; 1:10000, HRP-conjugated goat anti-mouse, BE0102; EASYBIO, China). The densitometric analysis of bands were performed with the Image J software and HSP90 was indicated as the internal reference.

For co-immunoprecipitation, dilute the samples to a protein concentration of 0.5-1 mg/mL. Take 600 μL of the sample and add 1 μg of Gal3-GFP protein. Mix well. Take 80 μL of the samples as input. Add 20 μL of Protein A/G Magnetic Beads (MJS002V2, MBL, Japan) and indicated antibodies to the remaining samples and operated following the instructions.

**Real-time PCR**

Total RNA of renal cells and mice kidneys were extracted with TRIZOL (DP424, TIANGEN Biotech, China). The mRNA was reverse transcripted into cDNA using a High-Capacity cDNA Reverse Transcription kit (4368813, Applied Biosystems, USA) and real-time RCR was carried out in a QuantStudio 3 Real-Time PCR system (Applied Biosystems, USA) with the SYBR Green qPCR Master Mix reagent (B21203, Bimake, USA). The relative mRNA expression levels of target genes were detected and analyzed via the ∆∆Ct method and the housekeeping gene *Rplp0* (*36B4*) was used as the internal reference. The primers of target genes were synthesized by RuiBiotech, China and the sequences were listed in Supplementary Table 3.

**Plasmid construction**

The point mutation plasmids of Pro-TGFβ1-His, TGFBR2-Myc and Ubiquitin-HA were constructed based on Pro-TGFβ1-His plasmid, TGFBR2-Myc plasmid and Ubiquitin-HA plasmid followed by the instructions of Fast Mutagenesis System kit (FM111-02, TransGen Biotech, China). Truncated Galectin-3 variants plasmids were constructed based on Galectin-3-His plasmid. The primers for point mutation plasmid construction were listed in Supplementary Table 4.

**Protein expression and purification**

Briefly, plasmids encoding truncated Galectin-3 variants (Galectin-3-His full, Galectin-3-His NSR, and Galectin-3-His CRD) were transformed into E. coli BL21(DE3) (CD601-02, TransGen Biotech, China) and cultured with IPTG induction for protein expression. After harvesting the bacterial cells, the target proteins were purified using a native His-tag protein purification kit (P2226, Beyotime Biotech, China) according to the manufacturer’s instructions.

**Bio-layer interferometry (BLI) assay**

Recombinant Gal3 and Pro-TGFβ1-His protein (50698-M08H, Sino Biological, China) were used. Pro-TGFβ1-His protein was captured by Ni-NTA (NTA) Biosensors (18-5101, Sartorius, Germany). Gal3 was diluted into gradient concentrations (500 nM, 250 nM, 125 nM, 62.5 nM, 31.25 nM, 17.13 nM and 0 nM). The equilibrium dissociation constant (*K*_D_) of Pro-TGFβ1-His with Gal3 in concentration gradient was measured by a BLI system with the Octet platform of ForteBio at 30 °C.

**Supplementary Figures:**


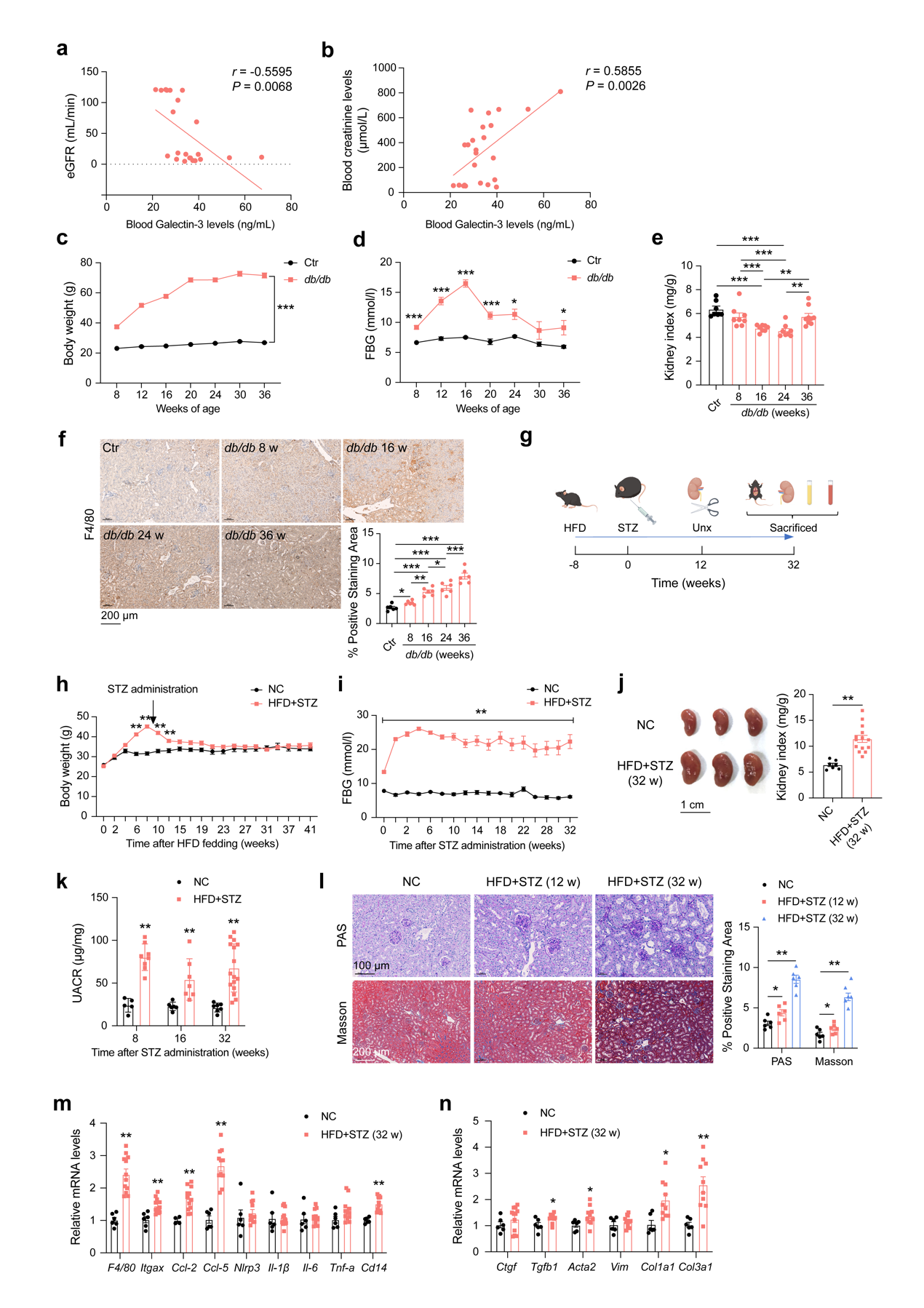


**Supplementary Figure S1. Increased inflammation and fibrosis during DKD progression.** **a-b**, Correlation between serum Gal3 levels and eGFR (**a**), and serum creatinine levels (**b**) in DKD patients. n = 22-24 patients. **c-e**, Body weight (**c**), fasting blood glucose (FBG) (**d**) and kidney index (**e**) of *db/db* mice at different weeks of age (n = 7-9 mice for each group). **f**, IHC staining of F4/80 in the kidney of *db/db* mice (n = 6 mice for each group, scale bar 200 μm). **g**, Schematic diagram of DKD model. **h-k**, Body weight (**h**), FBG (**i**), kidney index with representative images (**j**) and UACR (**k**) of DKD mice (n = 5-7 mice for NC, n = 7-16 mice for DKD). **l**, PAS and Masson staining of NC and DKD mice kidney (n = 6 mice for each group, scale bar 100 μm for PAS and 200 μm for Masson). **m-n**, mRNA expression levels of inflammation related genes (**m**) and fibrosis related genes (**n**) in NC and DKD mice kidney (n = 6 mice for NC, n = 10-12 mice for DKD). Data were analyzed by two-tailed Student’s t test and presented as the mean ± SEM. * *P* < 0.05; ** *P* < 0.01; *** *P* < 0.001; compared with control (NC) mice or indicated groups.


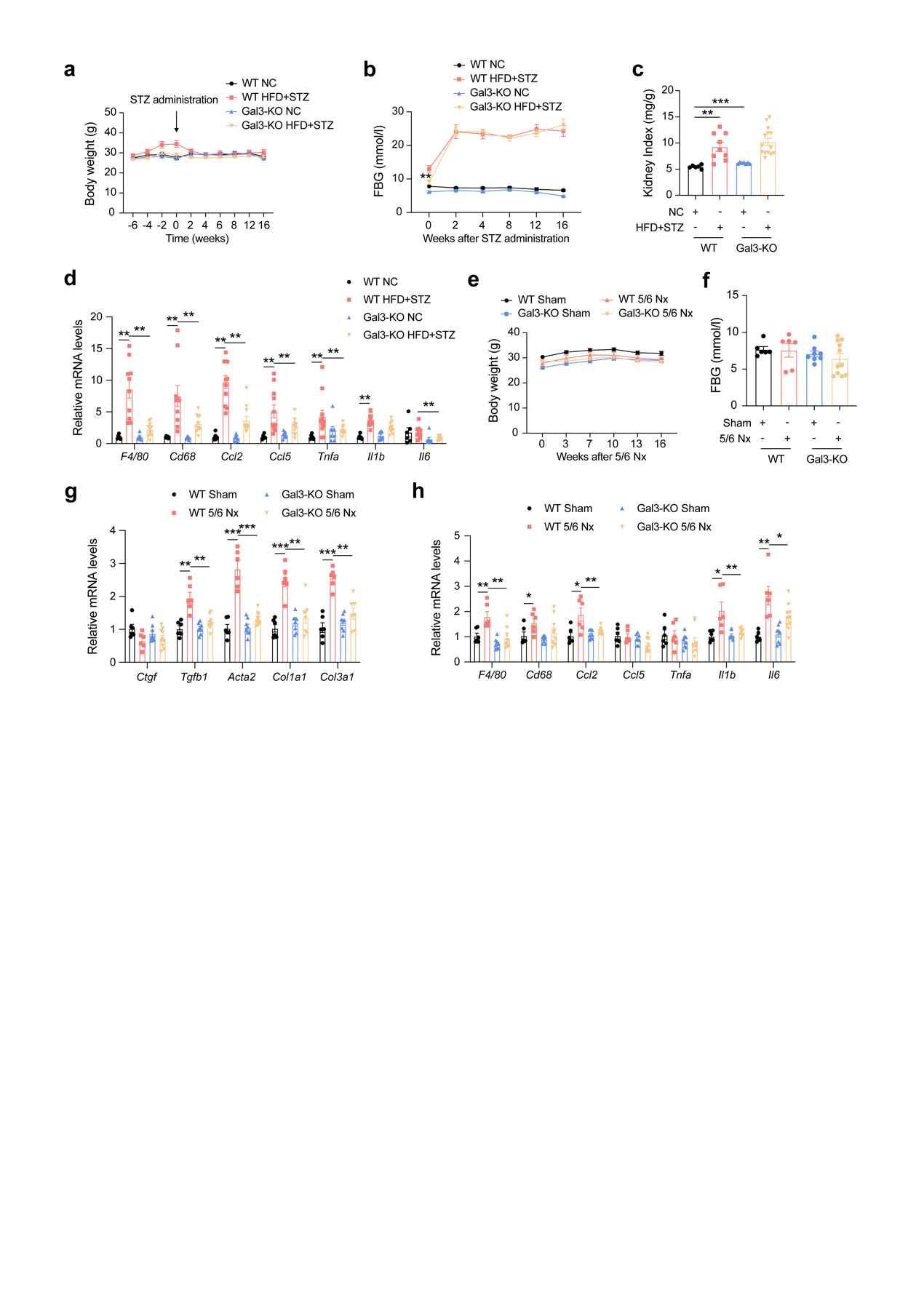


**Supplementary Figure S2.** **Gal3 ablation ameliorates kidney inflammation of DKD and CKD mice.** **a-c**, Body weight (**a**), FBG (**b**) and kidney index (**c**) in WT and Gal3-KO mice with or without DKD (n = 6-13 mice per group). **d**, Kidney mRNA expression levels of inflammation related genes (n = 6-11 mice per group). **e-f**, Body weight (**e**), FBG (**f**) of WT and Gal3-KO mice with or without CKD (n = 6-10 mice per group). **g-h**, Kidney mRNA expression levels of fibrosis (**g**) and inflammation (**h**) related genes (n = 6-10 mice per group). Data were analyzed by two-tailed Student’s t test and presented as the mean ± SEM. * *P* < 0.05; ** *P* < 0.01; *** *P* < 0.001; compared with WT HFD+STZ mice or indicated groups.


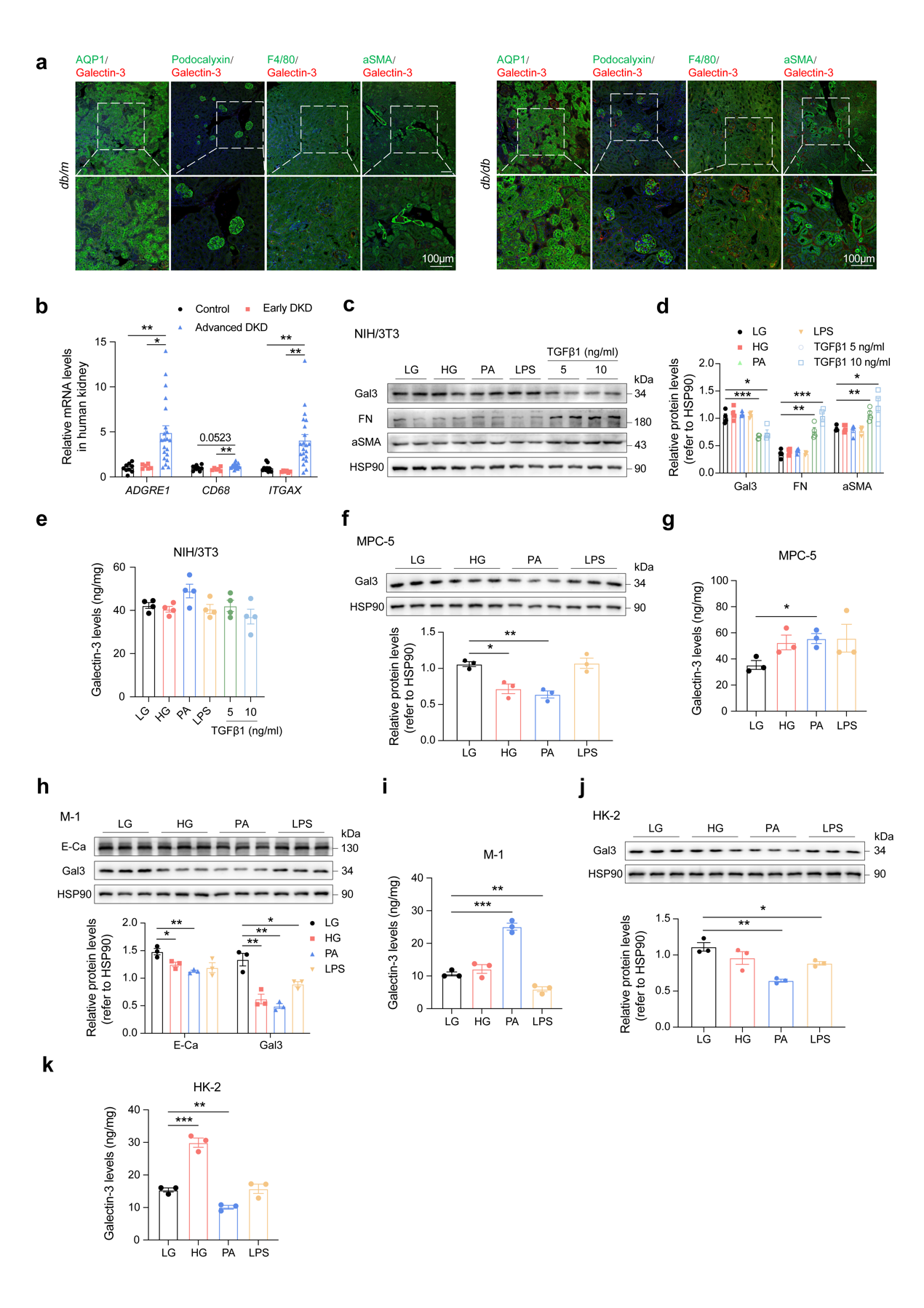


**Supplementary Figure S3. Gal3 expression and secretion in different kinds of kidney cells.** **a**, Kidney immunofluorescence co-staining of Gal3 (red) and other marker proteins (AQP1, Podocalyxin, F4/80, aSMA) (green) in *db/m* and *db/db* mice (**a**), Scale bar 100 μm. **b**, Human kidney mRNA levels of macrophage related genes (*ADGRE1*, *CD68* and *ITGAX*) in control, early DKD and advanced DKD patients (from GSE142025 dataset, n = 9 patients for control, n = 6 patients for early DKD, n = 21 patients for advanced DKD). **c-d**, Gal3 and FN and aSMA protein levels in NIH/3T3 fibroblasts after LG, HG, PA, LPS and TGFβ1 treatments (**c**) with statistical analysis (**d**). **e**, Gal3 levels in medium supernatant of NIH/3T3 fibroblasts after LG, HG, PA, LPS and TGFβ1 treatments. **f-g**, Gal3 levels in MPC-5 podocytes (**f**) and medium supernatant (**g**) after LG, HG, PA and LPS treatments. **h-i**, Gal3 and E-Ca protein levels in M-1 collecting duct cells (**h**) and medium supernatant (**i**) after LG, HG, PA and LPS treatments. **j-k**, Gal3 levels in HK-2 proximal tubular epithelial cells (**j**) and medium supernatant (**k**) after LG, HG, PA and LPS treatments. n = 4 independent cell samples per group (**c-e**) or n = 3 independent cell samples per group (**f-k**) for each experiment. LG: 5.56 mM glucose, HG: 30mM glucose, PA: 200 μM palmitic acid, LPS: 20 ng/mL lipopolysaccharide. Data were analyzed by two-tailed Student’s t test and presented as the mean ± SEM. * *P* < 0.05; ** *P* < 0.01; *** *P* < 0.001; compared with indicated groups.


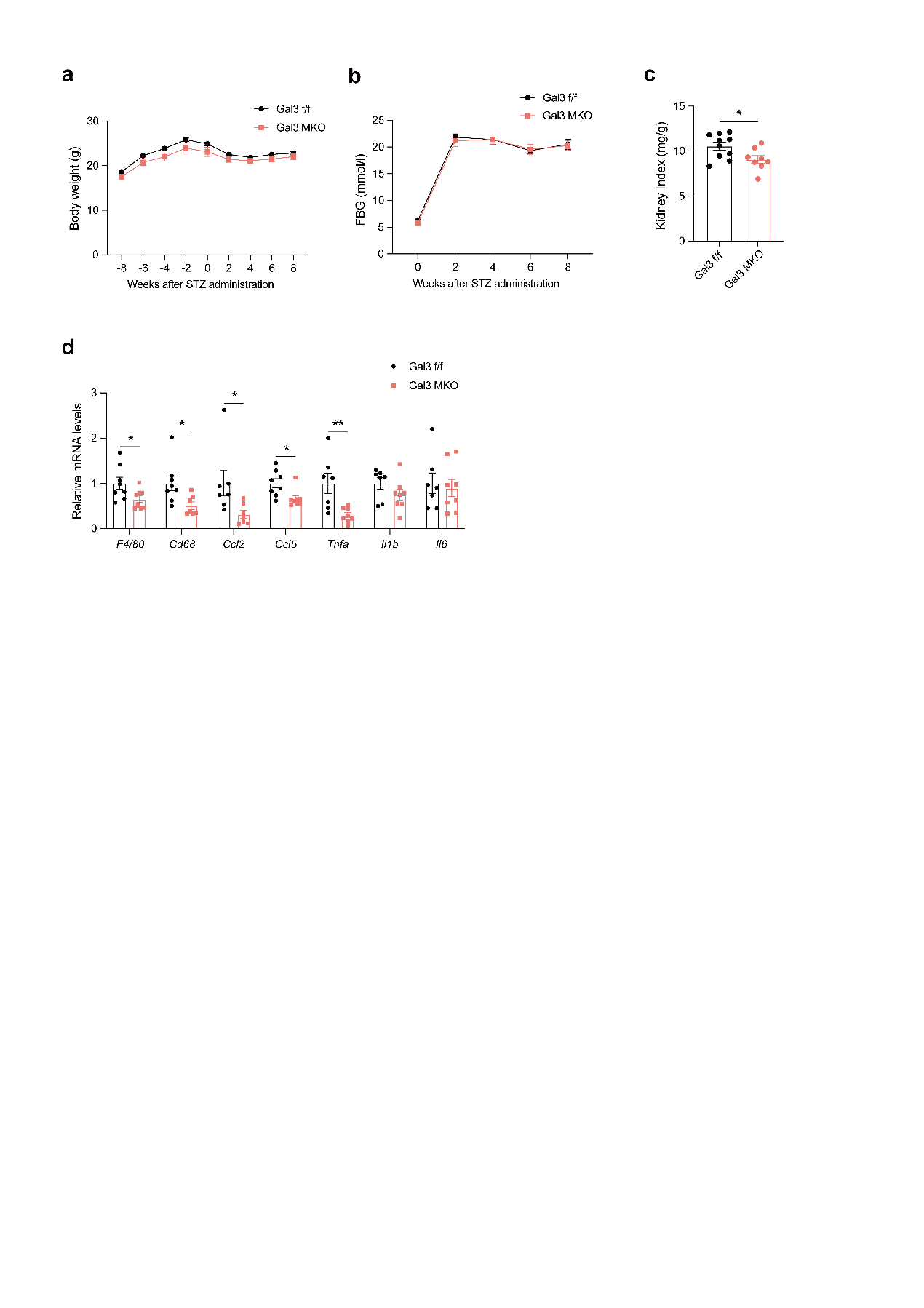


**Supplementary Figure S4. Gal3 specific knockout in macrophages ameliorates kidney inflammation of DKD mice. a-c**, Body weight (**a**), FBG (**b**) and Kidney index (**c**) of Gal3 f/f and Gal3 MKO mice with DKD. **d**, Lgals3 mRNA expression levels in macrophages, kidney, liver, EWAT and muscle in Gal3 f/f and Gal3 MKO mice with NC. **e**, kidney mRNA expression levels of inflammation related genes in Gal3 f/f and Gal3 MKO mice with DKD. n = 7-10 mice per group. Data were analyzed by two-tailed Student’s t test and presented as the mean ± SEM. * *P* < 0.05; ** *P* < 0.01; *** *P* < 0.001; compared with indicated groups.


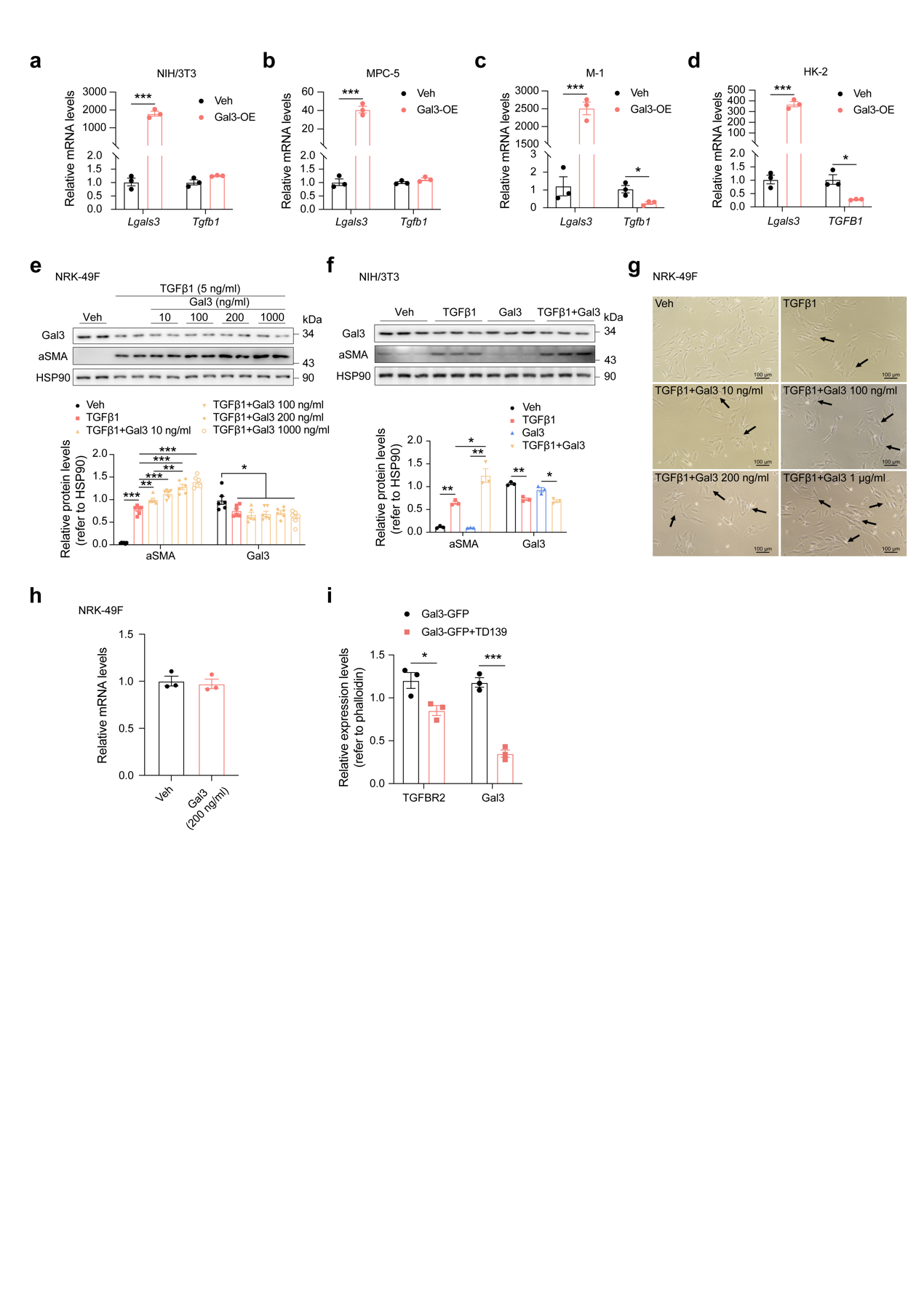


**Supplementary Figure S5. Gal3 does not regulate the expressions of Tgfb1 directly but enhances its pro-fibrotic effects.** **a-d**, *Lgals3* and *Tgfb1* mRNA levels after Gal3 overexpression in NIH/3T3 fibroblasts (**a**), MPC-5 podocytes (**b**), M-1 collecting duct cells (**c**) and HK-2 proximal tubular epithelial cells (**d**) (n = 3 independent cell samples per group for each experiment). **e**, Gal3 and aSMA protein levels in NRK-49F kidney fibroblasts after TGFβ1 and gradient Gal3 treatments (n = 6 independent cell samples per group). **f**, Gal3 and aSMA protein levels in NIH/3T3 fibroblasts after TGFβ1 and Gal3 treatments (n = 3 independent cell samples per group for each experiment). **g**, Representative images of NRK-49F fibroblasts after TGFβ1 and gradient Gal3 treatments, black arrow: activated fibroblasts. **h**, mRNA levels of *Tgfb1* in NRK-49F fibroblasts after Gal3 treatment (n = 3 independent cell samples per group for each experiment). **i**, Quantitative immunofluorescence analysis of figure 4i (n = 3 per group). Data were analyzed by two-tailed Student’s t test and presented as the mean ± SEM. * *P* < 0.05; ** *P* < 0.01; *** *P* < 0.001; compared with indicated groups.


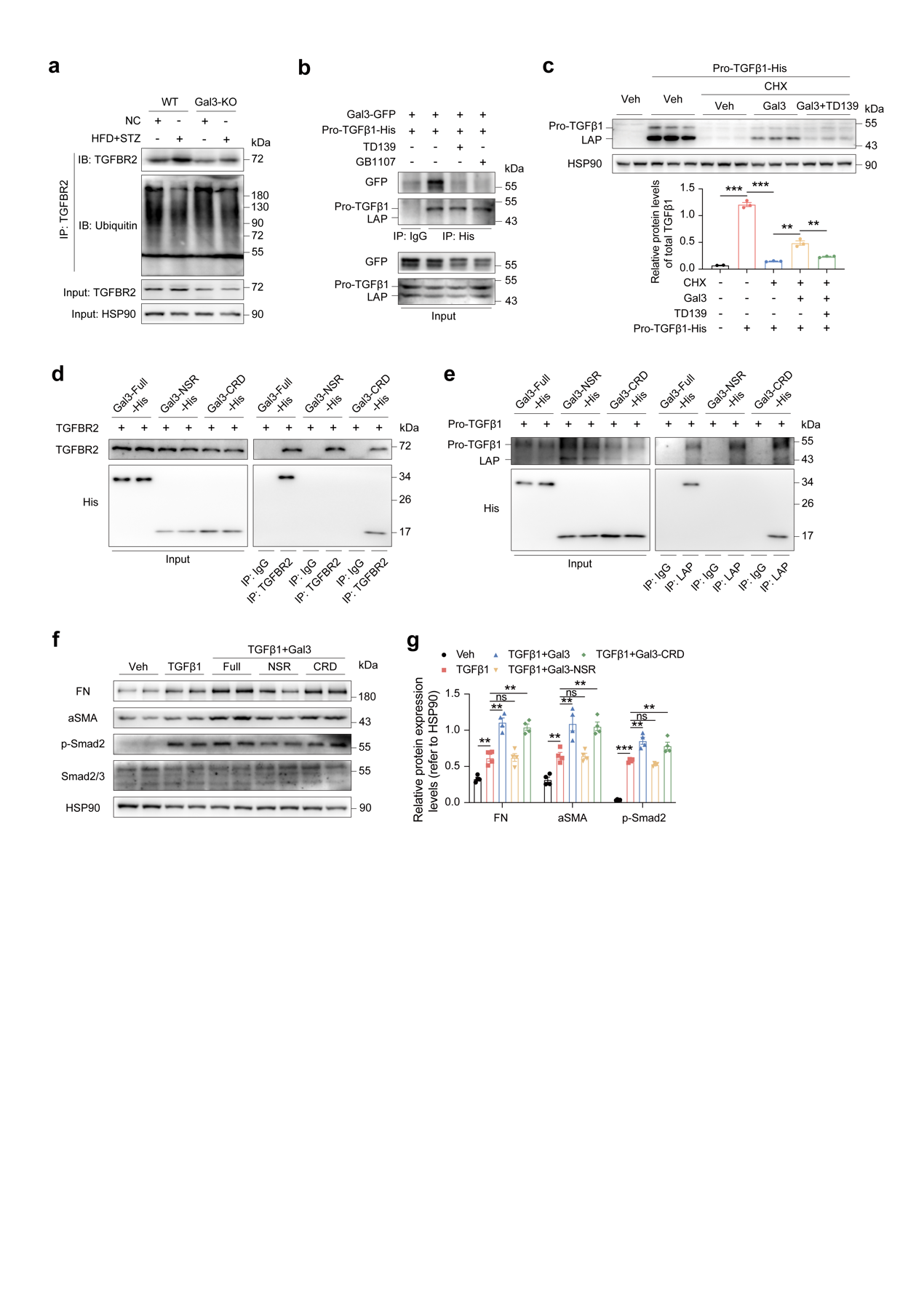


**Supplementary Figure S6. Gal3 promotes TGFβ1 signaling activation.** **a**, Gal3 knockout restored the ubiquitination of TGFBR2 in the kidney of DKD mice. **b.** Co-immunoprecipitation of Gal3-GFP and Pro-TGFβ1-His in 293T cells after Gal3 inhibitors TD139 and GB1107 treatments. **c**, Protein levels of Pro-TGFβ1 and LAP after Gal3, TD139 and CHX treatments in 293T cells with Pro-TGFβ1-His overexpression (n = 3 independent cell samples per group). **d**, The interactions between full length, NSR and CRD of Gal3 and TGFBR2. **e**, The interactions between full length, NSR and CRD of Gal3 and Pro-TGFβ1. **f**, CRD domain mediates Gal3 profibrotic effects in renal fibroblasts. **g**. Statistical analysis of (**f**) (n = 4 independent cell samples per group). Data were analyzed by two-tailed Student’s t test and presented as the mean ± SEM. ** *P* < 0.01; *** *P* < 0.001; compared with indicated groups. NSR (~112 amino acid), N-terminal with short end and repeat motif region; CRD (~130 amino acid), carbohydrate recognition domain.


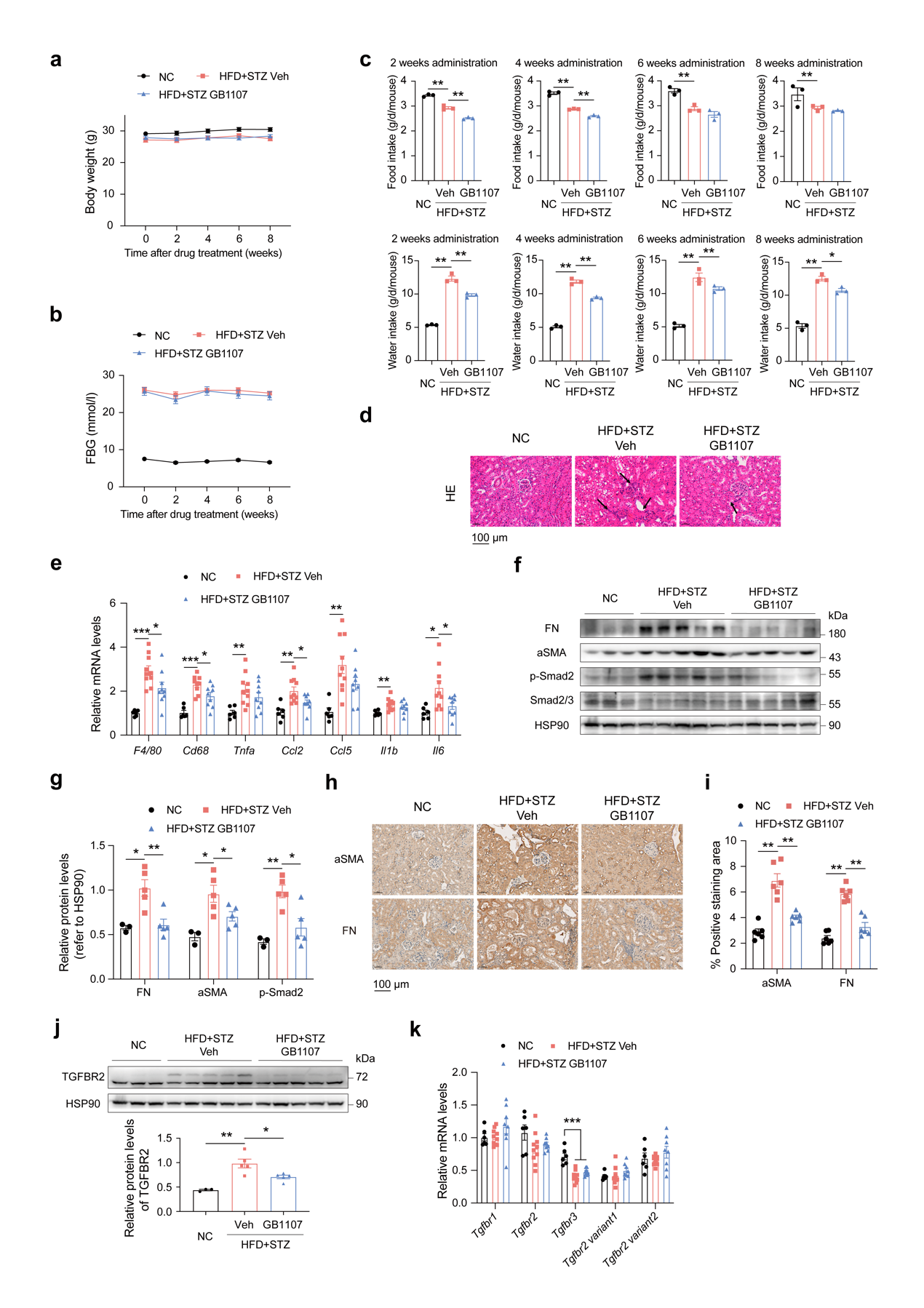


**Supplementary Figure S7. Pharmacological inhibition of Gal3 ameliorates diabetic symptoms and kidney fibrosis in DKD mice.** **a-b**, Body weight (**a**) and FBG (**b**) in NC, DKD mice treated with vehicle or GB1107. **c**, Food intake and water intake of DKD mice after different weeks of GB1107 treatment. n = 6-10 mice per group. **d**, HE staining of kidney in NC, DKD mice with vehicle or GB1107 treatment (n = 3 mice per group, scale bar 100 μm, black arrow: inflammatory cell infiltration). **e**, Kidney mRNA expression levels of inflammation related genes in NC, DKD mice with vehicle or GB1107 treatment (n = 6-10 mice per group). **f**, Protein levels of FN, aSMA, p-Smad2 and Smad2/3 in the kidney of NC, DKD mice with vehicle or GB1107 treatment (n = 3-5 mice per group). **g**, Statistical analysis of (**f**). **h**, IHC staining of α-SMA and FN in the kidney of NC, DKD mice with vehicle or GB1107 treatments (n = 6 mice per group, scale bar 100 μm). **i**, Statistical analysis of (**h**). **j**, Protein levels of TGFBR2 in the kidney of NC, DKD mice with vehicle or GB1107 treatment (n = 3-5 mice per group). **k**, Kidney mRNA expression levels of TGFβ1 receptors in NC, DKD mice with vehicle or GB1107 treatment (n = 6-10 mice per group). Data were analyzed by two-tailed Student’s t test and presented as the mean ± SEM. * *P* < 0.05; ** *P* < 0.01; *** *P* < 0.001; compared with indicated groups.

**Supplementary Tables:**

**Supplementary Table S1. Information of plasma donor patients**

| Group | Age (year) | Gender (Male/Female) | HbA1c  (%) | | eGFR  (mL/min) | Creatinine  (μmol/L) | Cystatin C  (mg/L) |
| --- | --- | --- | --- | --- | --- | --- | --- |
| DM | 50.44±14.09 | 18/11 | | 9.32±2.22 | 117.07±10.82 | 52.62±14.44 | 0.79±0.19 |
| DKD | 56.79±13.33 | 16/12 | | 8.12±3.72 | 50.83±49.75*** | 327.04±244.34*** | 2.78±1.67*** |
| Control | NA (Plasma of normal people are from physical examination center) | | | | | | |

Data were analyzed by two-tailed Student’s t test and presented as the mean ± SD. *** *P* < 0.001; compared with DM patients. DM: Diabetic patients without kidney disease; DKD: Diabetic kidney disease patients; NA: Not available.

**Supplementary Table S2. Information of kidney biopsy donor patients**

| Group | Age  (year) | Gender | FBG  (mM) | Creatinine  (μmol/L) | UACR  (μg/mg) |
| --- | --- | --- | --- | --- | --- |
| DKD | 66 | Female | 7.51 | 783.9 | 3965.63 |
| DKD | 34 | Female | 6.09 | 116.3 | 12890.46 |
| DKD | 53 | Female | 5.84 | 117.2 | 3538.54 |
| DKD | 64 | Male | 7.95 | 342.6 | 5381.91 |
| DKD | 60 | Male | 5.67 | 100.9 | 6509.51 |
| DKD | 62 | Male | 6 | 138.3 | 1712.76 |
| Control | NA (Control biopsy are from patients with minimal kidney lesion) | | | | |

DKD: Diabetic kidney disease patients; NA: Not available.

**Supplementary Table S3. Primer sequences for Real-time PCR**

| Gene | Sequence |
| --- | --- |
| Real-time PCR primer for mouse | |
| *Lgals3* Forward | AGACAGCTTTTCGCTTAACGA |
| *Lgals3* Reverse | GGGTAGGCACTAGGAGGAGC |
| *Ctgf* Forward | GGGCCTCTTCTGCGATTTC |
| *Ctgf* Reverse | ATCCAGGCAAGTGCATTGGTA |
| *Tgfb1* Forward | TAAAGAGGTCACCCGCGTGCTAAT |
| *Tgfb1* Reverse | ACTGCTTCCCGAATGTCTGACGTA |
| *Acta2* Forward | ATTGTGCTGGACTCTGGAGATGGT |
| *Acta2* Reverse | TGATGTCACGGACAATCTCACGCT |
| *Vimentin* Forward | CGGCTGCGAGAGAAATTGC |
| *Vimentin* Reverse | CCACTTTCCGTTCAAGGTCAAG |
| *Col1a1* Forward | GAGCGGAGAGTACTGGATCG |
| *Col1a1* Reverse | TACTCGAACGGGAATCCATC |
| *Col3a1* Forward | TCCTAACCAAGGCTGCAAGATGGA |
| *Col3a1* Reverse | ACCAGAATCTGTCCACCAGTGCTT |
| *F4/80* Forward | TGACTCACCTTGTGGTCCTAA |
| *F4/80* Reverse | CTTCCCAGAATCCAGTCTTTCC |
| *Itgax* Forward | CTGGATAGCCTTTCTTCTGCTG |
| *Itgax* Reverse | GCACACTGTGTCCGAACTCA |
| *Ccl2* Forward | TAAAAACCTGGATCGGAACCAAA |
| *Ccl2* Reverse | GCATTAGCTTCAGATTTACGGGT |
| *Ccl5* Forward | TTTGCCTACCTCTCCCTCG |
| *Ccl5* Reverse | CGACTGCAAGATTGGAGCACT |
| *Nlrp3* Forward | ATTACCCGCCCGAGAAAGG |
| *Nlrp3* Reverse | TCGCAGCAAAGATCCACACAG |
| *Cd14* Forward | CTCTGTCCTTAAAGCGGCTTAC |
| *Cd14* Reverse | GTTGCGGAGGTTCAAGATGTT |
| *Cd68* Forward | ACTTCGGGCCATGTTTCTCTT |
| *Cd68* Reverse | GGGGCTGGTAGGTTGATTGT |
| *Il-1β* Forward | TGGCAACTGTTCCTGAACTCAA |
| *Il-1β* Reverse | AGCAGCCCTTCATCTTTTGG |
| *Il-6* Forward | CTGCAAGAGACTTCCATCCAG |
| *Il-6* Reverse | AGTGGTATAGACAGGTCTGTTGG |
| *Tnfa* Forward | CAGGCGGTGCCTATGTCTC |
| *Tnfa* Reverse | CGATCACCCCGAAGTTCAGTAG |
| *Tgfbr1* Forward | TCTGCATTGCACTTATGCTGA |
| *Tgfbr1* Reverse | AAAGGGCGATCTAGTGATGGA |
| *Tgfbr2* Forward | GACTGTCCACTTGCGACAAC |
| *Tgfbr2* Reverse | GGCAAACCGTCTCCAGAGTAA |
| *Tgfbr2* variant1 Forward | CCGCTGCATATCGTCCTGTG |
| *Tgfbr2* variant1 Reverse | AGTGGATGGATGGTCCTATTACA |
| *Tgfbr2* variant2 Forward | TCCCAAGTCGGTTAACAGTGA |
| *Tgfbr2* variant2 Reverse | ACTTCATGCGGCTTCTCACA |
| *Tgfbr3* Forward | GGTGTGAACTGTCACCGATCA |
| *Tgfbr3* Reverse | GTTTAGGATGTGAACCTCCCTTG |
| *Rplp0* Forward | GCAGTCATAGCCACAGCATTT |
| *Rplp0* Reverse | AAGTGGAAAGCGAAGAATCG |
| Real-time PCR primer for rat | |
| *Lgals3* Forward | GCAACACGAAGCAGGACAATAACTG |
| *Lgals3* Reverse | CATCATTGACCGCAACCTTGAAGTG |
| *Acta2* Forward | GGCCGAGATCTCACCGACTA |
| *Acta2* Reverse | CACGCTCAGCAGTAGTCACG |
| *Tgfb1* Forward | CACTCCCGTGGCTTCTAGTG |
| *Tgfb1* Reverse | GGACTGGCGAGCCTTAGTTT |
| *Tgfbr1* Forward | GAAAGCATCGGCAAAGGTCG |
| *Tgfbr1* Reverse | CTGCCTCTCGGAACCATGAA |
| *Tgfbr2* Forward | CCCAAGTCGGTTAACAGCGA |
| *Tgfbr2* Reverse | TGTCGTTCTTCCTCCACACG |
| *Tgfbr3* Forward | CATCCCACCACATGATCCCG |
| *Tgfbr3* Reverse | AGGCGTTGATTGGTGACAGT |
| *Rplp0* Forward | AAGACTGGAGACAAGGTGGGA |
| *Rplp0* Reverse | TGGATGATCAGCCCGAAGGAGA |
| Real-time PCR primer for human | |
| *LGALS3* Forward | ATGGCAGACAATTTTTCGCTCC |
| *LGALS3* Reverse | GCCTGTCCAGGATAAGCCC |
| *TGFB1* Forward | CCCCTACATTTGGAGCCTGG |
| *TGFB1* Reverse | CACGTAGTACACGATGGGCA |
| *RPLP0* Forward | AGCCCAGAACACTGGTCTC |
| *RPLP0* Reverse | ACTCAGGATTTCAATGGTGCC |

**Supplementary Table S4. Primer sequences for point-mutation plasmid construction and truncated Galectin-3 variants**

| Plasmid | Sequence |
| --- | --- |
| Pro-TGFβ1 N53A Forward | CGGTGCTCGCTTTGTACGCCAGCACCCGCG |
| Pro-TGFβ1 N53A Reverse | GCGTACAAAGCGAGCACCGCCTCGGGCAGC |
| Pro-TGFβ1 N107A Forward | CACACAGTATATATATGTTCTTCGCTACGTCAGACATTCGG |
| Pro-TGFβ1 N107A Reverse | GCGAAGAACATATATATACTGTGTGAGATGTCTTTGG |
| Pro-TGFβ1 N147A Forward | AACTCTACCAGAAATATAGC GCCAATTCCTGGC |
| Pro-TGFβ1 N147A Reverse | GCGCTATATTTCTGGTAGAGTTCCACATGTTGCT |
| TGFBR2 N48A Forward | TCCATCCACCTAAGCTGTGCTAGGACCATCC |
| TGFBR2 N48A Reverse | GCACAGCTTAGGTGGATGGATGCATCTTTC |
| TGFBR2 N95A Forward | CAGAAGTCCTGCATGAGCGCCTGCAGCATCA |
| TGFBR2 N95A Reverse | GCGCTCATGCAGGACTTCTGGTTGTCGCAA |
| TGFBR2 N119A Forward | TGGAGGAAGAACGACAAGGCCATTACTCTGG |
| TGFBR2 N119A Reverse | GCCTTGTCGTTCTTCCTCCACACGGCCACG |
| Ubiquitin K48R Forward | TCATCTTTGCCGGCAGGCAGCTGGAA |
| Ubiquitin K48R Reverse | CTGCCGGCAAAGATGAGCCTCTGCT |
| Ubiquitin K63R Forward | ACTACAACATCCAGAGAGAGTCAACC |
| Ubiquitin K63R Reverse | CTCTGGATGTTGTAGTCAGAGAGGG |
| Galectin-3 Full Forward | CATGCCATGGCAGACAGCTTTTCGC |
| Galectin-3 Full Reverse | CCCAAGCTTGATCATGGCGTGGTTAGC |
| Galectin-3 NSR Forward | CATGCCATGGCAGACAGCTTTTCGC |
| Galectin-3 NSR Reverse | CCCAAGCTTGCCTCCAGGAGCACTGG |
| Galectin-3 CRD Forward | CATGCCATGGGCTATCCTGCTGCTGGC |
| Galectin-3 CRD Reverse | CCCAAGCTTGATCATGGCGTGGTTAGC |

**References**

1. Fan Y, Yi Z, D'Agati VD, et al. Comparison of Kidney Transcriptomic Profiles of Early and Advanced Diabetic Nephropathy Reveals Potential New Mechanisms for Disease Progression. *Diabetes*. 2019; 68: 2301-2314.

2. Furman BL. Streptozotocin-Induced Diabetic Models in Mice and Rats. *Curr Protoc*. 2021; 1: e78.

3. Jiang Q, Zhao Q, Chen Y, et al. Galectin-3 impairs calcium transients and β-cell function. *Nat Commun*. 2024; 15: 3682.

4. Du L, Chen Y, Shi J, et al. Inhibition of S100A8/A9 ameliorates renal interstitial fibrosis in diabetic nephropathy. *Metabolism*. 2023; 144: 155376.

5. Gao P, Li L, Yang L, et al. Yin Yang 1 protein ameliorates diabetic nephropathy pathology through transcriptional repression of TGFβ1. *Sci Transl Med*. 2019; 11(510): eaaw2050.
